# Azimuth-Frequency Conditioned Mamba U-Net for ISAR Image Refocusing

**DOI:** 10.3390/s26175419

**Published:** 2026-08-27

**Authors:** Shuge Wang, Wei Qu, Suqin Wu, Fusheng Wang, Bakun Zhu

**Affiliations:** Department of Electronic and Optical Engineering, Space Engineering University, Beijing 101416, China; quweistar@163.com (W.Q.); suqin_wu@hgd.edu.cn (S.W.); fusheng_wang@hgd.edu.cn (F.W.); zbk@hgd.edu.cn (B.Z.)

**Keywords:** inverse synthetic aperture radar, image refocusing, state-space model, Mamba architecture, skip connection

## Abstract

High-order phase defocus occurs in ISAR imaging of maneuvering targets. Traditional methods are limited by motion model mismatches, whereas existing deep imaging relies on complex-valued convolutions or self-attention mechanisms, whose excessive computational complexity hinders real-time processing. To address these issues, this paper proposes an Azimuth-Frequency Conditioned Mamba U-Net (AFC-Mamba U-Net) for ISAR image refocusing. The method treats the real and imaginary parts of the defocused complex image as dual-channel real-valued inputs, integrating linear-complexity state-space modeling with cross-layer feature fusion to significantly reduce computational and memory overhead while maintaining high refocusing quality. Specifically, we design an Azimuth-Frequency Conditional Mamba block (AFCMambaBlock), which performs bidirectional selective state-space scanning along the azimuth axis to capture long-range defocus dependencies with linear time complexity, and introduces azimuth-frequency conditional gating to incorporate frequency-domain phase error information into spatial features. A phase-state bottleneck is constructed to aggregate multi-scale enhanced states via a cross-scale state bank, forming globally phase-error-aware representations. Furthermore, we propose a state-guided skip connection mechanism that dynamically gates encoder skip features using bottleneck states, effectively suppressing the propagation of defocus artifacts while reducing redundant computations in the decoder. Experiments conducted on a simulated dataset containing eight types of spaceborne targets and real-world Yak-42 aircraft data demonstrate that the proposed method reduces MSE by 35.8% and NMSE by 41.5% compared to CNN-based U-Net, while achieving an SSIM of 0.9901. Compared to the state-of-the-art complex-domain network CVPHD, the proposed method achieves a significantly better efficiency–accuracy trade-off: it reduces model parameters and computational complexity by approximately two orders of magnitude, improves inference speed by more than fivefold, and cuts GPU memory usage by about 96%, while maintaining competitive structural consistency (SSIM > 0.99). Although CVPHD retains better MSE and NMSE on simulated data, the proposed method provides a lightweight and feasible solution for real-time and embedded ISAR refocusing.

## 1. Introduction

Inverse synthetic aperture radar is a well-established technique for obtaining high-resolution images of moving targets (ISAR) [1] and has been widely used in military and civilian applications. However, for non-cooperative maneuvering targets, the time-varying non-uniform rotational components induce migration through range cells (MTRC) and high-order phase errors, which severely defocus the resulting ISAR image. With the growing demand for fine-grained observation of space and airborne targets, on-orbit situational awareness, and continuous tracking of high-speed maneuvering targets, robust ISAR image refocusing has become essential for recovering high-resolution imagery.

Traditional ISAR refocusing methods mainly include non-parametric and parametric methods. Range instantaneous Doppler (RID) imaging [2] employs non-parametric time-frequency distributions, such as short-time Fourier transform (STFT), Wigner–Ville distribution (WVD) [2], and smoothed pseudo Wigner–Ville distribution (SPWVD) [3] to handle time-varying Doppler frequencies. However, RID methods suffer from cross-term interference when analyzing multi-component signals and offer only limited resolution. Parametric methods [4] fit the nonlinear phase errors caused by target rotation with a polynomial phase model (PPM) [5,6]. These methods achieve satisfactory compensation when the target motion is smooth, and the assumed model matches the actual motion; in practice, however, complex maneuvering targets often exhibit random jitter or high-order nonlinear motion components that lead to model mismatch, and the associated parameter estimation incurs a high computational cost.

To overcome these limitations, data-driven deep learning methods have emerged as a promising alternative for ISAR refocusing, with CNNs [7] and Transformers [8] demonstrating considerable potential. Early complex-valued deep neural networks (CV-DNNs) [9] and complex-valued convolutional neural networks (CV-CNNs) [10] adopt relatively simple architectures with single-scale convolutional kernels, which struggle to capture multi-scale scattering features ranging from fine details to coarse contours, thereby limiting their feature abstraction capability for complex target structures and high-order motion distortions. More recently, U-Net [11] and generative adversarial network (GAN) architectures have shown clear advantages in modeling the complex spatial dependencies of ISAR signals and images. Encoder–decoder architectures with specialized cross-layer fusion have also proven effective in related dense-prediction and multi-modal tasks [12], further motivating our U-Net-based design. Li et al. applied U-Net to wide-angle ISAR imaging and achieved fast focusing through complex-valued inputs and end-to-end training. The complex-valued GAN in [13] combines the generative capability of GANs with complex-domain operations, improving both the visual fidelity and the phase accuracy of the output images. Although CNN-based methods often incorporate elaborate attention mechanisms to extract informative features, they are inherently constrained by the limited receptive fields of convolutional units and thus cannot capture long-range dependencies. Transformer-based methods achieve global interactions among all input tokens through the self-attention mechanism, which enlarges the receptive field; however, their computational complexity grows quadratically with the number of tokens, posing severe challenges in high-resolution scenarios [14].

State-space models (SSMs), particularly Mamba [15] provide an efficient alternative for long-sequence modeling by achieving linear complexity through selective scanning, and Mamba has been successfully adapted to vision tasks such as image classification [16], image restoration [17], and frequency-domain processing [14,18].

To address the above issues, this paper introduces SSMs into ISAR image refocusing and proposes an Azimuth-Frequency Conditioned Mamba U-Net (AFC-Mamba U-Net). The network takes the real and imaginary parts of a defocused ISAR complex image as a two-channel real-valued input and performs end-to-end refocusing. Unlike generic vision Mamba architectures, the proposed network is specifically designed for the azimuth anisotropy and frequency-domain degradation characteristics of ISAR defocusing: (i) an AFCMambaBlock is embedded in the intermediate encoder stages to capture long-range azimuth dependencies with linear complexity; (ii) a phase-state bottleneck aggregates multi-scale defocus state features to construct a global phase-error-aware representation; and (iii) this state representation guides the skip connections to adaptively filter the defocus artifacts propagated from the encoder to the decoder. Together, these designs enable the network to effectively resolve azimuth defocusing caused by complex maneuvering while maintaining low computational overhead.

The main contributions of this paper are summarized as follows:

1. **Azimuth-Frequency Conditioned Mamba Block (AFCMambaBlock);**

ISAR image defocus arises primarily from azimuth phase errors and residual motion-compensation errors. To model the long-range coupling of defocus blur along the azimuth direction, we design a feature extraction module that integrates local convolution, bidirectional azimuth Mamba scanning, and azimuth-frequency conditional gating. By performing selective state-space scanning along the azimuth axis, the module captures long-range defocus dependencies with linear complexity, avoiding the redundant computation of global attention. Meanwhile, a one-dimensional spectral analysis along azimuth extracts the frequency-spreading features induced by phase errors and generates an adaptive gate that modulates the Mamba output, thereby embedding frequency-domain phase-error awareness into spatial-domain long-range modeling.

2. **Phase-State Bottleneck;**

To address azimuth defocusing caused by phase errors, we design a bottleneck that aggregates the multi-scale Mamba-enhanced state features of the encoder. By fusing azimuth long-range dependency features at different levels through a cross-scale state bank, a global state representation is constructed, which implicitly encodes the spatial distribution of phase errors in a data-driven manner and provides cross-layer semantic guidance for the decoder skip connections.

3. **State-Guided Skip Connection** **(SGSC);**

Since the skip paths of U-Net tend to propagate defocus artifacts, the bottleneck state is used to dynamically gate and filter the encoder skip features. This mechanism preserves high-resolution scattering-center details while suppressing artifacts carried by shallow defocused features, thereby improving the refocusing quality at the decoder side.

4. **Efficient ISAR Refocusing Network and Experimental Validation**.

Comparative and ablation experiments are conducted on a simulated space-target dataset and on real Yak-42 aircraft ISAR data. The proposed method significantly outperforms the traditional STFT method and the CNN-based baseline U-Net. Compared with the high-accuracy complex-valued network CVPHD, it maintains high structural consistency (SSIM > 0.99) while reducing the number of parameters by 99.3% and the computational complexity by 97.6%, improving the inference speed by more than 5×, and decreasing GPU memory usage by about 96%, making it more suitable for real-time and embedded deployment.

## 2. Methodology

This section first establishes the ISAR echo signal model under non-cooperative target maneuvering and analyzes the physical mechanism and spatially varying characteristics of azimuth defocusing. On this basis, the Azimuth-Frequency Conditioned Mamba U-Net (AFC-Mamba U-Net) is presented, including its overall architecture, core modules (AFCMambaBlock, phase-state bottleneck, and state-guided skip connection), and the design of the jointly supervised loss function.

### 2.1. ISAR Echo Signal Modeling

**Figure 1:**
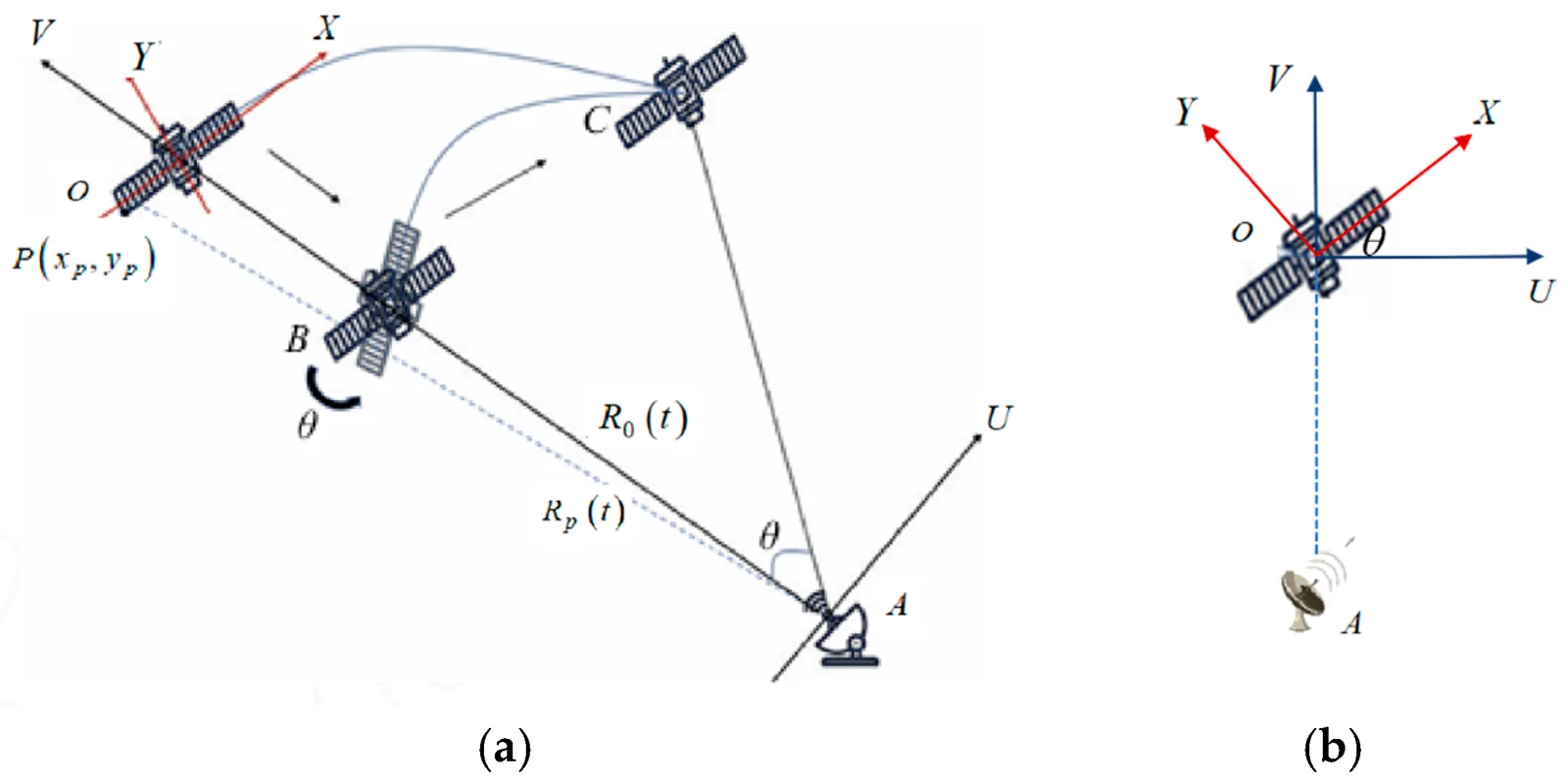
Schematic of ISAR target motion model: (**a**) schematic of the relative motion between radar and target; (**b**) reference imaging coordinate system.

In ISAR imaging, the motion of a target relative to the radar can be decomposed into translation and rotational components. Figure 1 shows a schematic of the relative motion between the radar and the target. The coordinate plane is defined by the satellite trajectory and the radar phase center, where A denotes the radar observation station, O denotes the target center, and the radar line of sight points from A to O.

As shown in Figure 1b, the satellite, as the observed target, rotates relative to the radar. XOY is the observation coordinate system fixed to the radar, and UOV is the target coordinate system fixed to the satellite, with the target rotating about point O. $R_{0}$ denotes the distance from the target reference center to the radar. The observation angle θ is the angle between the target and radar coordinate systems, whose variation reflects the relative rotation between the target and the radar.

Assume $\theta_{0}$ is the initial angle between the target coordinate system and the radar coordinate system, $\omega_{\theta }$ and $\alpha_{\theta }$ are the initial angular velocity and angular acceleration of the target rotation, respectively, and $t_{m}$ denotes the slow time; then:

(1) $$\theta =\theta_{0}+\omega_{\theta }t_{m}+\frac{1}{2}\alpha_{\theta }t_{m}^{2}$$

The correspondence between the UOV coordinate system and the XOY coordinate system is:

(2) $$\left\{\begin{array}{l} u=x\cos\theta -y\sin\theta \\ v=x\sin\theta +y\cos\theta \end{array}\right.$$

Let the coordinates of the p-th scattering point of the target in the target coordinate system be $\left(x_{p},y_{p}\right)$; the instantaneous distance from this point to the radar is then:

(3) $$\begin{array}{l} R_{rp}\left(t_{m}\right)=\sqrt{{\left[R_{0}\left(t_{m}\right)+v\left(t_{m}\right)\right]}^{2}+u{\left(t_{m}\right)}^{2}}\\ =\sqrt{R_{0}{\left(t_{m}\right)}^{2}+v{\left(t_{m}\right)}^{2}+u{\left(t_{m}\right)}^{2}+2R_{0}\left(t_{m}\right)\cdot v\left(t_{m}\right)}\\ =\sqrt{R_{0}{\left(t_{m}\right)}^{2}+x{\left(t_{m}\right)}^{2}+y{\left(t_{m}\right)}^{2}+2R_{0}\left(t_{m}\right)\cdot v\left(t_{m}\right)}\\ =\sqrt{R_{0}{\left(t_{m}\right)}^{2}+x{\left(t_{m}\right)}^{2}+y{\left(t_{m}\right)}^{2}+2R_{0}\left(t_{m}\right)\cdot \left[x\left(t_{m}\right)\sin\theta \left(t_{m}\right)+y\left(t_{m}\right)\cos\theta \left(t_{m}\right)\right]} \end{array}$$

When the radar–target distance is much larger than the target size, Equation (3) can be approximated as:

(4) $$R_{rp}\left(t_{m}\right)\approx R_{0}\left(t_{m}\right)+x\left(t_{m}\right)\sin\theta \left(t_{m}\right)+y\left(t_{m}\right)\cos\theta \left(t_{m}\right)$$

According to its contribution to imaging, $R_{rp}\left(t_{m}\right)$ can be decomposed into $R_{0}\left(t_{m}\right)$ and $R_{p}\left(t_{m}\right)$, where $R_{0}\left(t_{m}\right)$ represents the motion common to all scattering points of the target and $R_{p}\left(t_{m}\right)$ is the component directly related to imaging.

A linear frequency modulated (LFM) signal is used as the radar transmitted signal, expressed as Equation (5):

(5) $$s\left(\hat{t};t_{m}\right)=rect\left(\frac{\hat{t}}{T_{P}}\right)\cdot \exp\left[j2\pi \left(f_{c}t+\frac{1}{2}\gamma {\hat{t}}^{2}\right)\right]$$

where $\hat{t}\in \left[-T_{P}/2,T_{P}/2\right]$ is the fast time, $T_{P}$ is the pulse width, $t_{m}\in \left[-T_{a}/2,T_{a}/2\right]$ is the slow time, $T_{a}$ is the coherent processing interval (CPI), $\gamma =B/T_{P}$ is the chirp rate of the LFM signal, B is the bandwidth, $f_{c}$ is the carrier frequency, $t=t_{m}+\hat{t}$ is the full time, and rect() denotes the rectangular function. Assuming that the target consists of P scattering points, the received echo signal can be expressed as:

(6) $$s_{e}\left(\hat{t};t_{m}\right)=\sum_{p=1}^{P}\sigma_{p}rect\left(\frac{\hat{t}-\frac{2R_{rp}\left(t_{m}\right)}{c}}{T_{P}}\right)\cdot \exp\left[j2\pi \left(f_{c}\left(t-\frac{2R_{rp}\left(t_{m}\right)}{c}\right)+\frac{1}{2}\gamma {\left(\hat{t}-\frac{2R_{rp}\left(t_{m}\right)}{c}\right)}^{2}\right)\right]$$

where $\sigma_{p}$ is the reflection coefficient of the p-th scattering point.

Using the dechirp-on-receive mode, after removing the residual video phase (RVP) that causes imaging blur and performing velocity compensation, the range profile of the signal can be obtained via Fourier transform pulse compression, expressed as:

(7) $$s_{ve}\left(t_{e};t_{m}\right)=\sum_{p=1}^{P}\sigma_{p}\sin c\left[B\cdot \left(t_{e}-\frac{2R_{rp}\left(t_{m}\right)}{c}\right)\right]\exp\left[-j2\pi f_{c}\frac{2R_{rp}\left(t_{m}\right)}{c}\right]$$

Owing to the time–frequency equivalence of the LFM signal, $t_{e}$ provides an intuitive representation of the range distribution. After translational motion compensation to eliminate $R_{0}\left(t_{m}\right)$, the compensated range profile is denoted as [19]:

(8) $$s_{p}\left(t_{e};t_{m}\right)=\sum_{p=1}^{P}\sigma_{p}\sin c\left[B\cdot \left(t_{e}-\frac{2R_{p}\left(t_{m}\right)}{c}\right)\right]\exp\left[-j2\pi f_{c}\frac{2R_{p}\left(t_{m}\right)}{c}\right]$$

where, from Equation (8), $R_{p}\left(t_{m}\right)$ can be expressed as:

(9) $$R_{p}\left(t_{m}\right)=x_{p}\cos\theta \left(t_{m}\right)+y_{p}\sin\theta \left(t_{m}\right)$$

Performing Taylor series expansion of the trigonometric functions, $R_{p}\left(t_{m}\right)$ is approximated as:

(10) $$R_{p}\left(t_{m}\right)\approx x_{p}\left(1-\frac{\theta {\left(t_{m}\right)}^{2}}{2!}\right)+y_{p}\theta \left(t_{m}\right)$$

Substituting Equation (1) into Equation (10),

(11) $$R_{p}\left(t_{m}\right)=R_{p0}+\upsilon_{p}t_{m}+a_{p}t_{m}^{2}+b_{p}t_{m}^{3}+c_{p}t_{m}^{4}$$

and

(12) $$\begin{array}{l} R_{p0}=x_{p}+y_{p}\theta_{0}-\frac{x_{p}\theta_{0}^{2}}{2},\\ \upsilon_{p}=\omega_{\theta }\left(y_{p}-x_{p}\theta_{0}\right),\\ a_{p}=\frac{1}{2}\left[\alpha_{\theta }y_{p}-x_{p}\left(\omega_{\theta }^{2}+\theta_{0}\alpha_{\theta }\right)\right],\\ b_{p}=-\frac{x_{p}\omega_{\theta }\alpha_{\theta }}{2},c_{p}=-\frac{x_{p}\alpha_{\theta }^{2}}{8} \end{array}$$

Substituting Equation (12) into Equation (8) yields Equation (13), which contains terms of all orders together with the spatially varying terms that may cause migration through range cells and phase errors [20].

(13) $$\begin{array}{l} S_{p}\left(t_{e},t_{m}\right)=\sum_{p=1}^{P}\sigma_{p}\sin c\left\{B\cdot \left[t_{e}-\frac{2}{c}\left(R_{p0}+\upsilon_{p}t_{m}+a_{p}t_{m}^{2}+b_{p}t_{m}^{3}+c_{p}t_{m}^{4}\right)\right]\right\}\\ \cdot \exp\left[\frac{-j4\pi f_{c}}{c}\left(R_{p0}+\upsilon_{p}t_{m}+a_{p}t_{m}^{2}+b_{p}t_{m}^{3}+c_{p}t_{m}^{4}\right)\right] \end{array}$$

As indicated by Equation (13), the high-order phase terms introduced by target rotation remain after translational motion compensation and cause residual azimuth phase errors in the echo of each scattering point. These high-order terms spread the azimuth Doppler spectrum, preventing the scattering points from being precisely focused and thereby defocusing the ISAR image in azimuth. Moreover, since the high-order phase coefficients of each scattering point depend directly on its cross-range coordinate $\left(x_{p},y_{p}\right)$, the azimuth defocusing is markedly spatially variant. Meanwhile, because these high-order phase terms are continuous functions of the slow time, their influence extends over the entire CPI, giving rise to pronounced long-range coupling of the azimuth defocusing.

### 2.2. Design Motivation and Overall Architecture

The signal model and the physical characteristics analyzed in Section 2.1 impose three requirements on the network design: (1) the long-range coupling of azimuth defocusing requires the network to model long-range context along the azimuth direction so as to capture phase-error correlations across slow-time samples; (2) the spatial variability of the defocusing calls for multi-scale global aggregation to handle position-dependent blur; and (3) the spectral spreading induced in the azimuth-frequency domain by high-order phase errors should be exploited to assist refocusing.

To this end, this paper proposes the Azimuth-Frequency Conditioned Mamba U-Net (AFC-Mamba U-Net), which adopts an encoder-bottleneck-decoder structure. While ISAR signals are intrinsically complex-valued, all existing Mamba implementations are strictly real-valued and lack native complex-domain support. The proposed network therefore takes the real and imaginary parts of the defocused ISAR image as dual-channel real-valued inputs and compensates for the missing phase coupling through azimuth-frequency conditional gating, which implicitly exploits phase-error-induced spectral spreading. End-to-end ISAR refocusing is achieved through the combination of azimuth long-range state-space modeling, frequency-domain feature assistance, and multi-scale global state aggregation.

**Figure 2:**
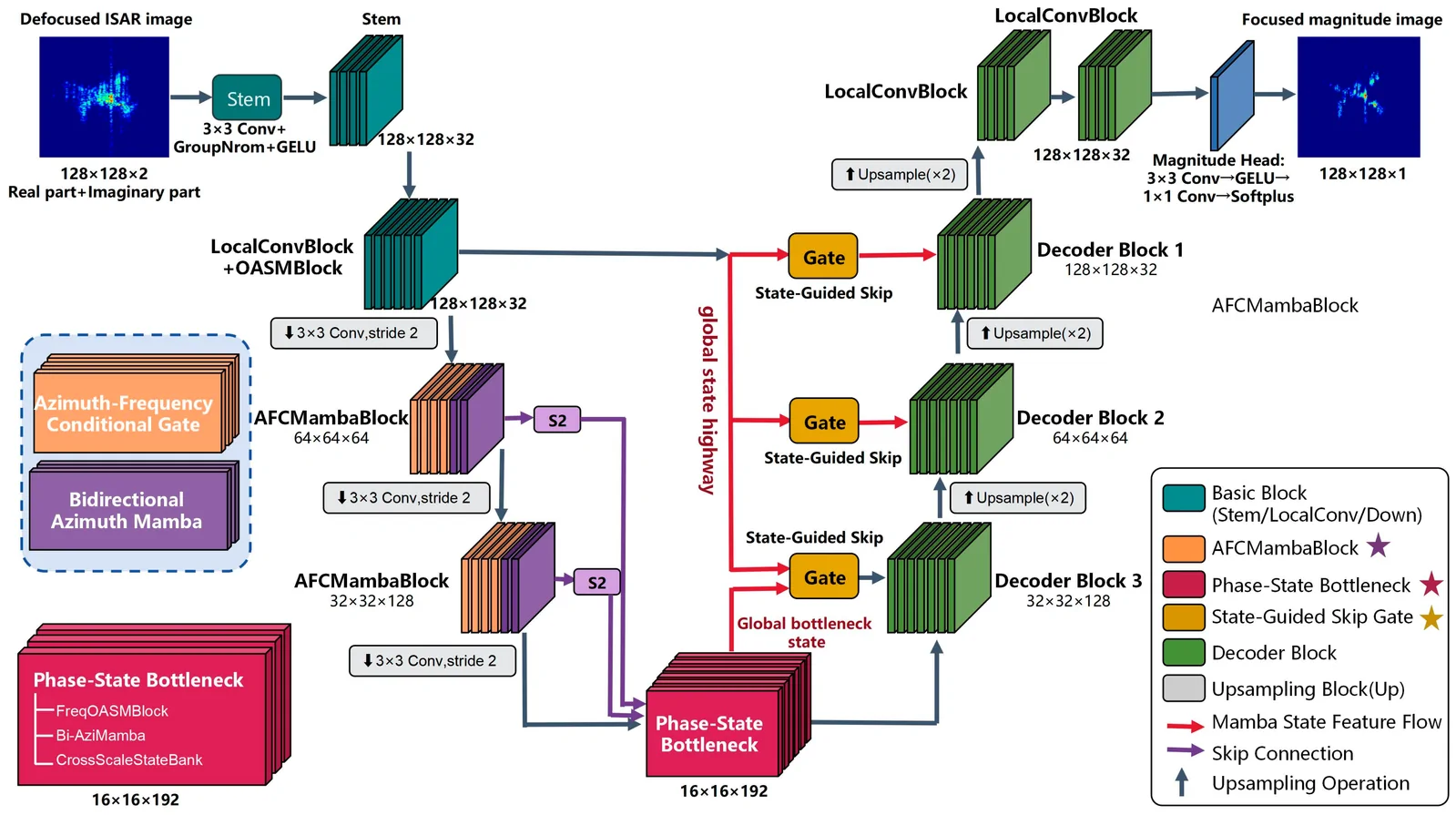
Overall architecture of the AFC-Mamba U-Net. The network takes dual-channel real-valued inputs (the real and imaginary parts of the defocused image) and outputs the focused magnitude image. The first encoder stage employs local convolution (LocalConvBlock + OASMBlock); the second and third stages employ AFCMambaBlocks; the phase-state bottleneck aggregates multi-scale state features; and the decoder uses state-guided skip connections.

As shown in Figure 2, the first encoder stage uses a LocalConvBlock and an OASMBlock to extract low-level scattering-center features. The second and third stages employ AFCMambaBlocks, which output refined feature maps together with Mamba-enhanced state features. After three downsampling operations, the deepest features are processed by the phase-state bottleneck, which aggregates the Mamba-enhanced states from the second and third encoder stages into a global bottleneck state. The decoder progressively restores the spatial resolution through upsampling blocks, state-guided skip connections, and local convolution blocks. Finally, the magnitude head predicts the focused amplitude image.

### 2.3. Specific Implementation of the Module

#### 2.3.1. AFCMambaBlock

**Figure 3:**
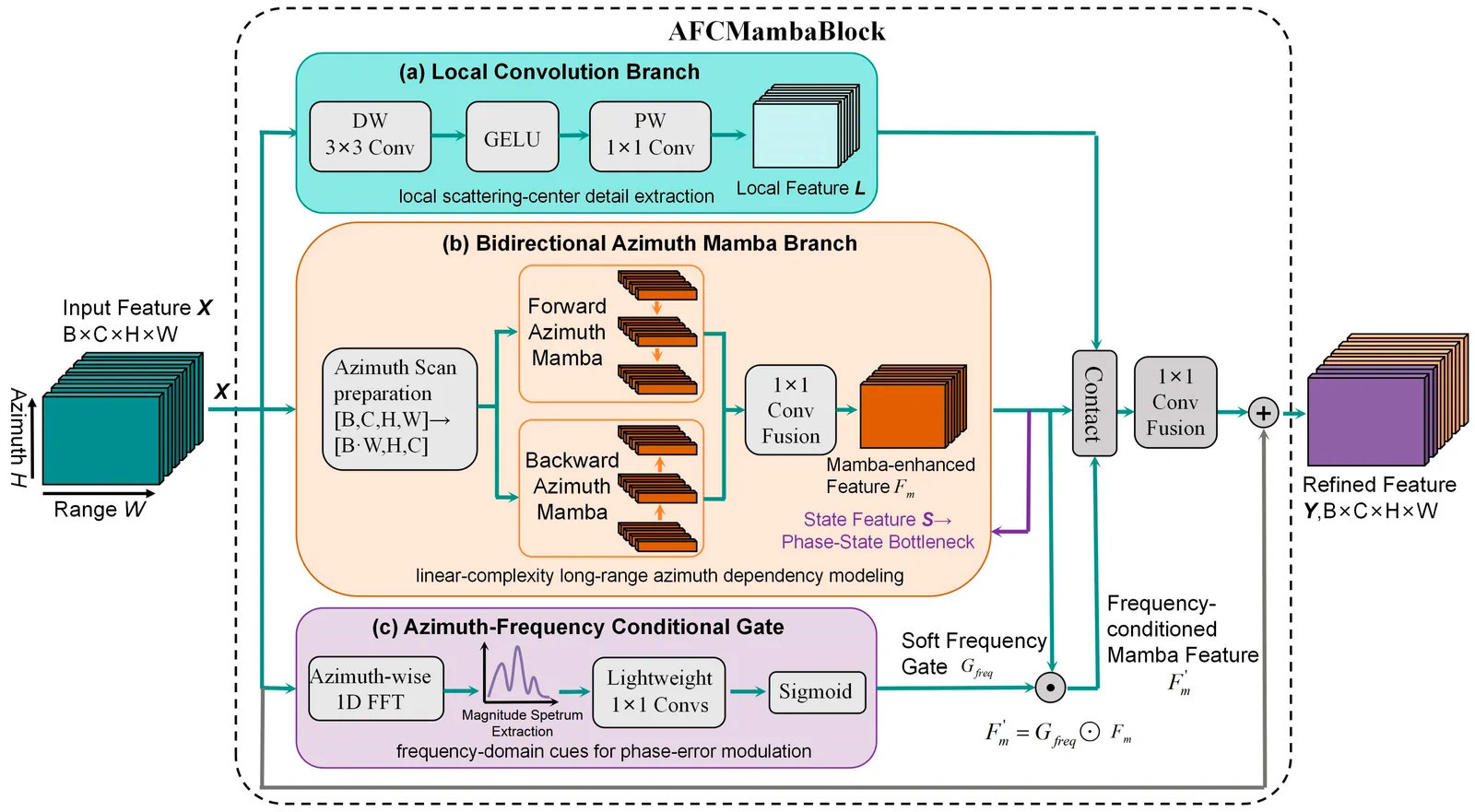
Detailed structure of the AFCMambaBlock. The module consists of three branches: (**a**) a local convolution branch that captures short-range spatial details; (**b**) a bidirectional azimuth Mamba branch that models long-range azimuth dependencies; and (**c**) an azimuth-frequency conditional gate that perceives phase-error-induced spectral spreading and generates a soft gate to adaptively modulate the Mamba output. The modulated Mamba features are concatenated and fused with the local features, and the refined feature Y is produced through a residual connection.

Since phase errors caused by target maneuvering mainly disrupt azimuth coherent integration and thereby defocus the ISAR image in azimuth, we forgo generic global attention and instead perform selective state-space modeling along the azimuth axis, resulting in the AFCMambaBlock. The module consists of three parts: a local convolution branch, a bidirectional azimuth Mamba branch, and an azimuth-frequency conditional gate, see Figure 3.

**Local convolution branch:** Given the input feature $X\in R^{B\times C\times H\times W}$, the local branch extracts short-range spatial details:

(14) $$F_{l}=Conv_{1\times 1}\left(GELU\left(DWConv_{3\times 3}\left(X\right)\right)\right)$$

A depthwise separable 3 × 3 convolution captures the local spatial context around each scattering point in a lightweight manner, and a 1 × 1 pointwise convolution maps the local details into a feature space compatible with the Mamba branch, preparing the features for the subsequent cross-branch fusion with the long-range azimuth dependency features.

**Bidirectional azimuth Mamba branch:** To model long-range dependencies efficiently, the feature map is reshaped, for each range bin, into a sequence of length H along the azimuth dimension:

(15) $$X_{a}\in R^{\left(BW\right)\times H\times C}$$

Here, W and H correspond to the range (fast-time) and azimuth (slow-time) axes, respectively. Forward and backward selective state-space scans are then applied to capture contextual information from both azimuth directions:

(16) $$F_{f}={Mamba}_{f}\left(X_{a}\right),F_{b}=Flip\left({Mamba}_{b}\left(Flip\left(X_{a}\right)\right)\right)$$

After reshaping the scan outputs back to the two-dimensional feature space, the features from both directions are fused through a 1 × 1 convolution:

(17) $$F_{m}=Fuse\left(\left[F_{f},F_{b}\right]\right)$$

This design enables the network to model long-range azimuth dependencies with linear computational complexity $O\left(H\right)$, instead of the quadratic complexity incurred by global attention.

**Azimuth-frequency conditional gate:** To further extract frequency-domain feature information, a one-dimensional real-valued fast Fourier transform (FFT) is performed along the azimuth direction on the group-normalized features, and the amplitude spectrum is extracted:

(18) $$A=\left|rFFT\left(GN\left(X\right),\dim=H\right)\right|$$

where $GN\left(\cdot \right)$ represents group normalization. Owing to the conjugate symmetry of the real-valued FFT, this yields H/2+1 independent frequency components, which are then projected back to length H by a learned 1 × 1 convolution to restore the original spatial size. A subsequent 1 × 1 convolution and Sigmoid activation produce the soft frequency gate $G\in R^{C\times H\times W}$:

(19) $$G_{f}=\sigma \left(Conv_{1\times 1}\left(A\right)\right)$$

Since $G\in R^{C\times H\times W}$ is a dense three-dimensional tensor in which each element is an independent scalar, the gate acts simultaneously as a channel-wise weighting mechanism and as a spatially varying modulator. This gate adaptively modulates the Mamba-enhanced features:

(20) $${\tilde{F}}_{m}=F_{m}⊙\left(1+\left|\alpha \right|\cdot G_{f}\right)$$

where α is a learnable scaling parameter initialized to a small value to suppress gradient perturbations in the early training stage. This frequency-conditioning mechanism performs adaptive modulation in the spatial domain: the azimuth spectral distribution is used to characterize the degree of frequency spreading caused by phase errors, and the response weights of the spatial-domain Mamba features are adjusted accordingly.

In ISAR imaging, the phase of a defocused image is highly sensitive to arbitrary initial pulse phases and residual translational compensation errors, making it unreliable for stable feature extraction. The amplitude spectrum, in contrast, provides a translation-invariant and physically interpretable measure of phase-error-induced frequency spreading.

**Residual fusion and output:** To combine local spatial details with long-range azimuth features, the local feature $F_{l}$ and the frequency-gated Mamba-enhanced feature ${\tilde{F}}_{m}$ are concatenated along the channel dimension and fused by a 1 × 1 convolution. The fused features are weighted by a learnable scaling parameter γ and added to the input feature X through a residual connection, yielding the refined feature map:

(21) $$Y=X+\left|\gamma \right|\cdot Conv_{1\times 1}\left(\left[F_{l},{\tilde{F}}_{m}\right]\right)$$

The AFCMambaBlock outputs two features: the refined feature map Y and the Mamba-enhanced state feature ${\tilde{F}}_{m}$. The latter encodes the long-range contextual dependencies captured by the Mamba branch along the azimuth direction and is referred to as the state feature in this paper. Serving as a task-level latent representation for ISAR refocusing, this state feature is used for cross-scale state aggregation in the phase-state bottleneck and for decoder feature purification in the state-guided skip connections.

#### 2.3.2. Phase-State Bottleneck

The high-order phase errors remaining after translational compensation act mainly along the azimuth direction, causing image defocusing with long-range azimuth coupling. Unlike traditional methods that explicitly estimate polynomial phase coefficients, the phase-state bottleneck implicitly learns the spatial distribution of phase errors in a data-driven manner, aggregating multi-scale encoder context into a global state representation to guide decoder skip connections.

The bottleneck comprises three components: a frequency-enhanced OASM block (FreqOASMBlock), a bidirectional azimuth Mamba module (BiAziMamba), and a cross-scale state bank. Given the deepest encoder feature $X_{4}$, the processing flow is as follows.

**Frequency-enhanced OASM block:** This block combines local depthwise convolution with a frequency-domain processing branch. The local convolution stabilizes the deep feature representation in the spatial domain, while the frequency-domain branch maps the features to the frequency domain via a two-dimensional fast Fourier transform (2-D FFT), adaptively processes the spectral coefficients through learnable convolutions, and maps them back to the spatial domain via an inverse FFT (IFFT). The deepest encoder feature $X_{4}\in R^{B\times C\times H\times W}$ is further enhanced after joint spatial-frequency processing by the FreqOASM block:

(22) $$F_{p}=FreqOASM\left(X_{4}\right)$$

To prevent the numerical perturbations introduced by the frequency-domain branch from disrupting spatial-domain feature learning, a frequency-gating mechanism constrains the response amplitude of the frequency-domain branch with a learnable scaling factor. This allows the network to exploit phase-error-induced frequency-domain structural information for deep feature enhancement while preserving a robust spatial-domain representation.

**Bidirectional azimuth Mamba**: By scanning the feature map forward and backward along the azimuth direction, the module efficiently encodes global contextual dependencies with linear computational complexity. The bidirectional azimuth Mamba is applied to the frequency-refined features to further capture long-range azimuth dependencies:

(23) $$F_{b}=BiAziMamba\left(F_{p}\right)$$

**Cross-scale state bank:** To aggregate azimuth long-range context from different encoder levels and thereby strengthen the global representation of the bottleneck, the Mamba-enhanced state features $S_{2}$ and $S_{3}$ output by the second and third encoder stages are projected to the bottleneck channel dimension and resampled to the bottleneck resolution:

(24) $${\bar{S}}_{i}=\mathrm{Resize}\left({\mathrm{Proj}}_{i}\left(S_{i}\right)\right),i\in \left\{2,3\right\}$$

The projected state features are concatenated along the channel dimension and fused via convolution to generate a global state representation:

(25) $$S_{g}=Fuse\left(\left[{\bar{S}}_{2},{\bar{S}}_{3}\right]\right)$$

**Global fusion and residual output:** The global state is fused with the Mamba-enhanced bottleneck feature and projected back to the bottleneck dimension:

(26) $$S_{B}=Proj\left(\left[F_{b},S_{g}\right]\right)$$

**Figure 4:**
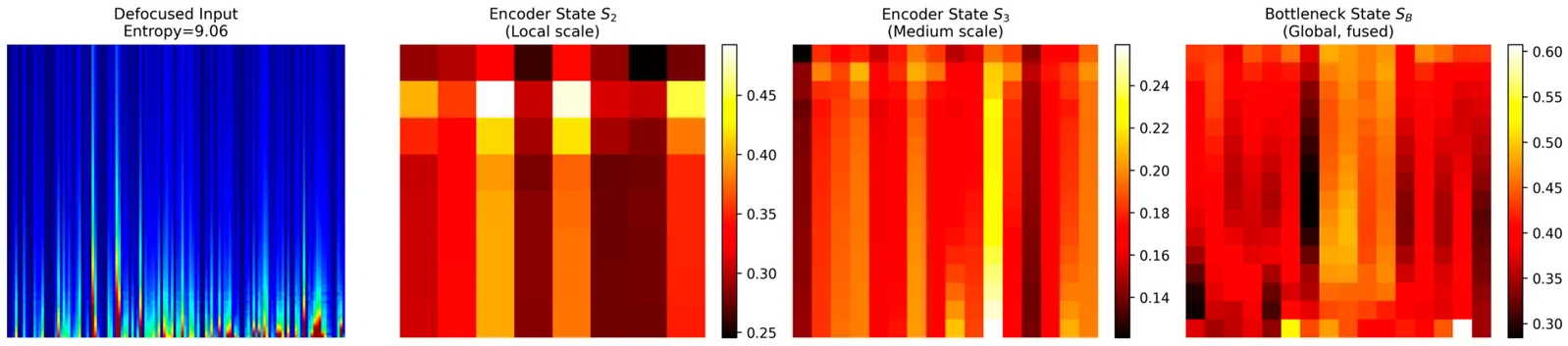
Multi-Scale Phase-State Aggregation (CrossScaleStateBank). Multi-scale state aggregation for a severely defocused sample. The color intensity from blue (low) to red/yellow (high) represents the magnitude of the respective state values. $S_{2}$ (local) shows block-wise activation, $S_{3}$ (medium) exhibits transitional vertical textures, and $S_{B}$ (global, fused) forms distinct vertical stripe structures aligned with the azimuth blur direction. The colorbars indicate the value ranges for each encoder/bottleneck state.

The final bottleneck output is obtained through a residual connection with a learnable scaling parameter β:

(27) $$B=X_{4}+\left|\beta \right|\cdot S_{B}$$

where $S_{B}$ serves as the bottleneck global state transmitted to the decoder skip connections. Unlike explicit polynomial phase compensation methods that rely on parametric error estimation, the proposed bottleneck learns the representation of phase-error-induced defocusing in an implicit, task-adaptive manner, thereby providing robust cross-scale guidance during decoding. Figure 4 presents the spatial distribution and the statistical behavior of the learned states on the validation set.

As shown in Figure 4, the encoder states $S_{2}$ and $S_{3}$ capture azimuth long-range dependencies at local and medium scales, respectively. $S_{2}$ exhibits block-wise activation patterns corresponding to local defocus details, while $S_{3}$ begins to show vertical textures aligned with the azimuth direction. After aggregation by the CrossScaleStateBank, the bottleneck state $S_{B}$ forms distinct vertical stripe structures that are globally consistent with the azimuth smearing direction of the severely defocused input. This multi-scale evolution confirms that $S_{B}$ is not a passive encoder feature but a phase-error-aware representation emergent from cross-scale state fusion.

**Figure 5:**
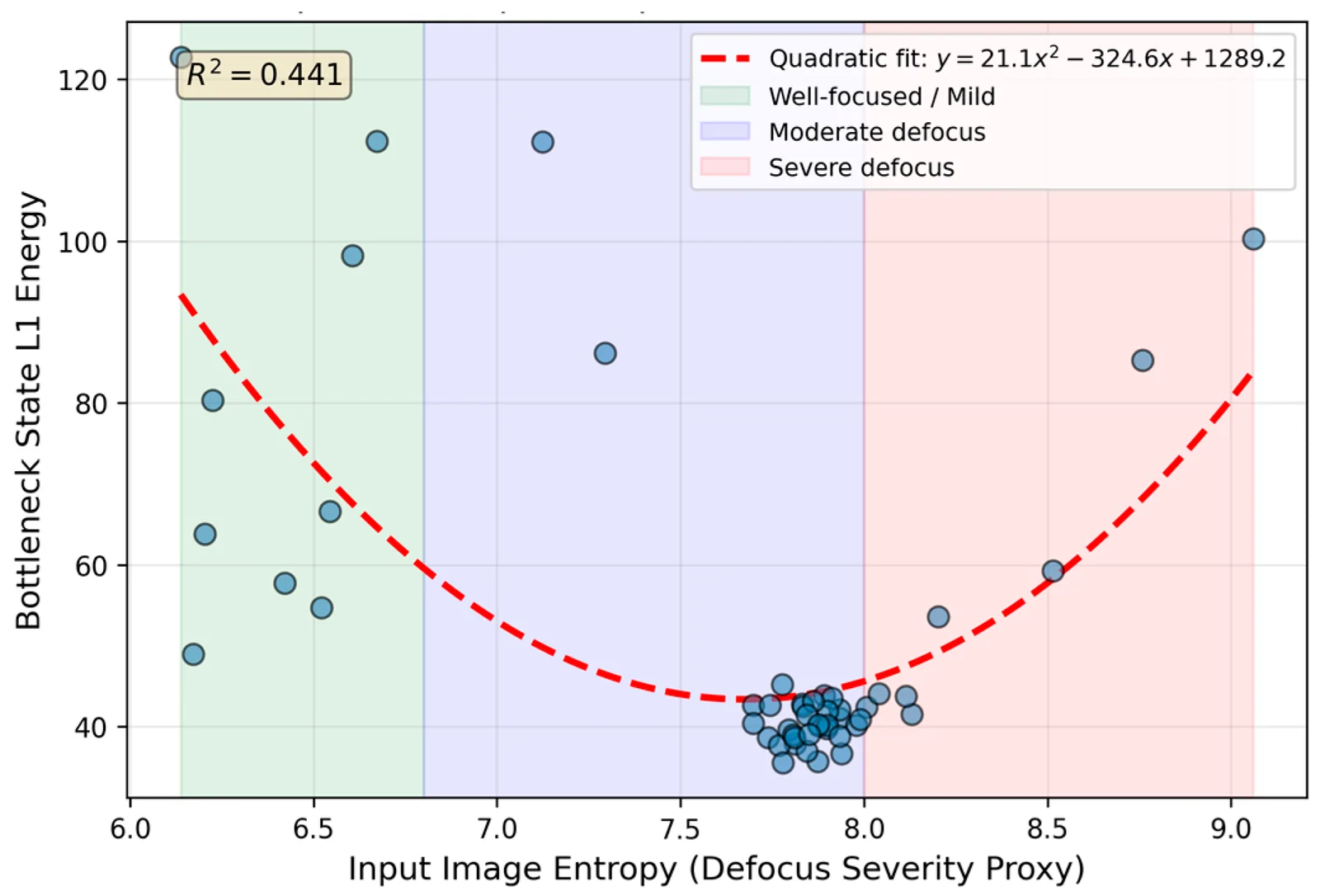
Adaptive response of phase-state bottleneck. U-shaped dependence of bottleneck state energy on input image entropy ($R^{2}=0.441$), demonstrating the adaptive, defocus-dependent nature of the phase-state bottleneck. Each blue dot represents an individual test sample.

Quantitatively, as shown in Figure 5, the bottleneck state energy exhibits a U-shaped dependence on the input defocus severity (quantified by the image entropy), with a quadratic fit yielding $R^{2}=0.441$. This non-monotonic, adaptive response demonstrates that the phase-state bottleneck acts as a task-specific regulator.

#### 2.3.3. State-Guided Skip Connection

**Figure 6:**
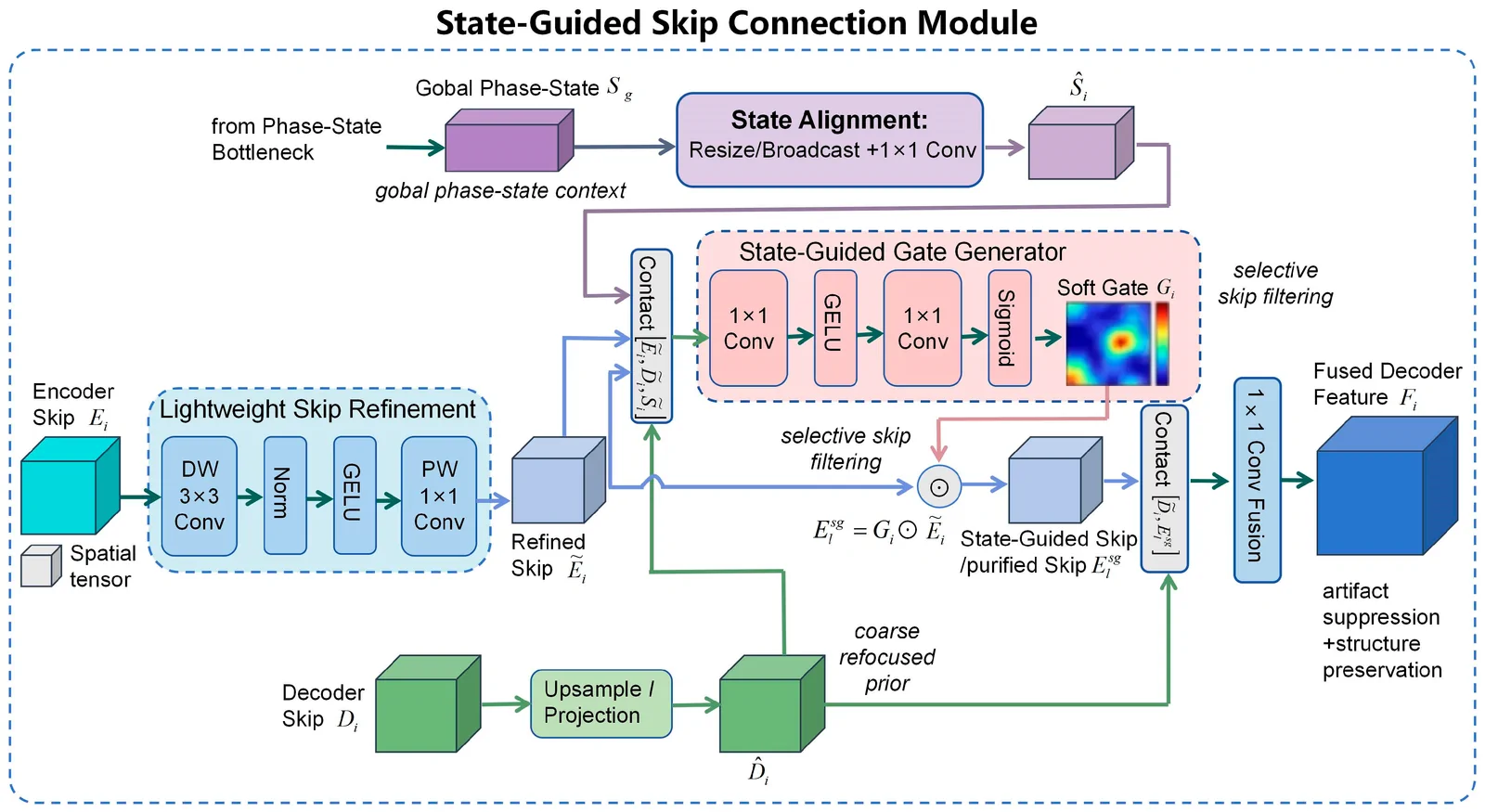
Detailed structure of the state-guided skip connection. The encoder features are first processed by an Anti-Artifact Block and a refinement convolution, while the bottleneck state is projected and resized to the skip resolution. A gate is then generated from the refined encoder features, the decoder features, and the projected state to adaptively control the skip information flow.

Skip connections are essential for preserving high-resolution spatial details in U-Net-like architectures. In ISAR refocusing, however, shallow encoder features inevitably contain defocus smearing, sidelobe artifacts, and azimuth blur; propagating these features directly to the decoder may reintroduce degradation patterns and degrade the refocusing quality. To address this issue, we propose the state-guided skip connection (SGSC), which introduces the bottleneck global state as high-level semantic guidance to achieve adaptive purification and selection of skip features, see Figure 6.

**Encoder feature purification:** The encoder features are first passed through an Anti-Artifact Block to suppress potential defocusing smears and sidelobe artifacts in shallow features. This block employs lightweight depthwise convolutions constrained by a small residual scaling factor, which attenuates degradation patterns while preserving the spatial locations of scattering centers. A Refine Convolution then further refines the feature representation:

(28) $$E_{r}=\mathrm{Refine}\left(AntiArtifact\left(E\right)\right)$$

**Bottleneck state projection:** To enable global phase-error-aware information to guide feature selection in skip connections, the bottleneck state $S_{B}$ is projected to the channel dimension of the skip features and resized to the same spatial resolution:

(29) $$S_{r}=\mathrm{Resize}\left({\mathrm{Proj}}_{s}\left(S_{B}\right)\right)$$

**Adaptive gate generation:** The purified encoder features $E_{r}$, decoder features D, and projected bottleneck state $S_{r}$ are concatenated, and a spatially adaptive gate map is generated through 1 × 1 convolution and Sigmoid activation:

(30) $$G_{s}=\sigma \left(Conv\left(\left[E_{r},D,S_{r}\right]\right)\right)$$

Guided by the global bottleneck state, this gating mechanism evaluates the information quality of the encoder features at each spatial location: gate values close to 1 preserve useful details, whereas values close to 0 suppress artifacts.

**Gated fusion and output:** The gate map adaptively weights the purified encoder features, which are then concatenated with the decoder features and compressed to produce the output of this skip connection:

(31) $$Z=Compress\left(\left[D,G_{s}\right.\right.⊙\left.\left.E_{r}\right]\right)$$

With this design, the SGSC allows the decoder to adaptively select useful spatial details from the encoder under the guidance of the global bottleneck state while explicitly suppressing defocus smearing and artifact contamination. Compared with unguided direct skip connections, this mechanism effectively blocks the propagation of degradation patterns between the encoder and decoder, significantly improving refocusing quality.

#### 2.3.4. Loss Function

The network is trained with a multi-task joint supervision strategy in which spatial-domain reconstruction serves as the main constraint, complemented by frequency-domain consistency and structure-aware regularization terms. Given the network-predicted focused amplitude image $\hat{Y}$ and its corresponding ground-truth focused amplitude image Y, the total loss function consists of five weighted terms:

1. Amplitude Loss (pixel-level fidelity constraint):

(32) $$L_{mag}={\left\|\hat{Y}-Y\right\|}_{1}$$

This loss directly constrains the pixel-level accuracy of the reconstructed image. Compared with the $L_{2}$ loss, the $L_{1}$ loss is less sensitive to outliers and can produce sharper reconstruction results, which is beneficial for preserving the focused structure of scattering points in ISAR images and avoiding the inherent over-smoothing effect of the $L_{2}$ loss.

2. Logarithmic Amplitude Loss (dynamic range stability constraint):

(33) $$L_{\log}={\left\|\log\left(1+\hat{Y}\right)-\log\left(1+Y\right)\right\|}_{1}$$

ISAR images exhibit significant amplitude differences between strong and weak scattering points. The logarithmic transformation compresses the amplitude dynamic range, so that this term preserves the fidelity of strong scattering points while also accounting for weak scattering points and background regions, preventing the network from over-optimizing strong-scatterer regions at the expense of weak ones.

3. Gradient Loss (edge structure preservation constraint):

(34) $$L_{grad}={\left\|\nabla \hat{Y}-\nabla Y\right\|}_{1}$$

where ∇ denotes the spatial gradient operator. By enforcing consistency between the gradient distributions of the predicted and ground-truth images, this term enhances the recovery of scattering-point edges and local structures and suppresses the scattering-point spreading and boundary blurring caused by over-smoothing.

4. Dual-Domain Loss (frequency-domain consistency constraint):

(35) $$L_{dual}={\left\|F\left(\hat{Y}\right)-F\left(Y\right)\right\|}_{1}$$

where $F\left(\cdot \right)$ denotes the two-dimensional fast Fourier transform operator. This term directly supervises the frequency-domain matching and thus ensures global spectral consistency.

5. Scattering Loss (focusing-clarity constraint):

(36) $$L_{scatter}={\left\|\sigma \left(\hat{Y}\right)-\sigma \left(Y\right)\right\|}_{1}$$

where $\sigma \left(\cdot \right)$ denotes the image standard deviation computed over a local window. By constraining the local contrast matching between the predicted and ground-truth images, this term enhances the focusing clarity of scattering centers, encouraging high-contrast peak structures in the reconstructed image and avoiding excessive energy spreading.

The final total loss function is the weighted combination of the above five terms:

(37) $$L_{total}=\lambda_{mag}L_{mag}+\lambda_{\log}L_{\log}+\lambda_{grad}L_{grad}+\lambda_{dual}L_{dual}+\lambda_{scatter}L_{scatter}$$

The amplitude and gradient losses serve as the main constraints, while the logarithmic, dual-domain, and scattering losses act as auxiliary regularizers. These terms work synergistically and prevent the network from sacrificing spatial-domain pixel accuracy by overfitting frequency-domain statistics.

## 3. Experiments and Results

### 3.1. Experimental Dataset

We evaluate the proposed method on both simulated and real ISAR datasets. The simulated dataset is the publicly available dataset constructed in [13], in which ISAR images are generated with an ideal point-scattering model for eight categories of space targets: NSS-7, KH-4, Tstar-1, FM5, KH-12, Insat-1, MS-19, and CZ-1. High-order phase errors are introduced into the echo signals to simulate defocusing, and the radar parameters are configured according to the Gaofen-3 satellite (Table 1). Each category contains 200 images, which are split into 190 training and 10 test samples with a fixed random seed (seed = 42) to ensure reproducibility. Complex-valued ISAR images are fed into the network as two-channel inputs using their real and imaginary parts, with the corresponding focused amplitude images serving as ground truth. The images are cropped into 128 × 128 patches via a sliding-window strategy with a stride of 64 pixels along both the range and azimuth directions. For loss-weight selection, the coefficients were determined according to the relative contribution scale of each term: the amplitude and gradient losses directly constrain pixel-level reconstruction fidelity and edge preservation, and are treated as the primary supervision terms ($\lambda_{mag}=1.0$, $\lambda_{grad}=0.3$); the logarithmic loss stabilizes the dynamic range of strong and weak scattering responses ($\lambda_{\log}=0.5$); the dual-domain and scattering losses serve as auxiliary regularizers for frequency-domain consistency and local scattering sharpness ($\lambda_{dual}=0.1$, $\lambda_{\mathrm{scatter}}=0.01$). These weights were validated through the loss-term ablation and sensitivity analysis experiments reported in Section 3.4.

For the real ISAR dataset, we used the maneuvering flight segments of the Yak-42 aircraft. Since no ground-truth focused image is available for the real data, we conduct mainly qualitative evaluations, complemented by no-reference focusing metrics, including the image entropy and the image contrast.

### 3.2. Experimental Setup and Evaluation Indicators

#### 3.2.1. Experimental Setup

All experiments are conducted on a platform equipped with an NVIDIA RTX 4090 GPU (NVIDIA Corporation, Santa Clara, CA, USA), and the network is implemented in PyTorch 2.3.X with Python 3.10 and CUDA 11.8. The channel dimensions of the four scales are set to 32, 64, 128, and 192, respectively. The Mamba state dimension is set to 16 and the expansion ratio to 2. The model is trained with the Adam optimizer at an initial learning rate of 2 × 10^−4^ and a batch size of 4 for 120 epochs.

#### 3.2.2. Evaluation Indicators

To conduct a multi-dimensional and objective quantitative assessment of the ISAR refocusing results, this paper mainly employs the following indicators to evaluate the refocusing performance from five aspects: pixel reconstruction accuracy, structural fidelity, energy concentration, scattering clarity, and computational efficiency.

1. Mean Squared Error (MSE) [21];

The MSE measures the average intensity deviation between corresponding pixels of the reconstructed image and the reference image (the perfectly focused image) and is the most fundamental metric of pixel-level reconstruction accuracy. For an image with dimensions M × N, let the reconstructed image be I and the reference image be K. The MSE is defined as:

(38) $$\mathrm{MSE}=\frac{1}{\mathrm{MN}}\sum_{i=0}^{M-1}\sum_{j=0}^{N-1}{\left[I\left(i,j\right)-K\left(i,j\right)\right]}^{2}$$

The smaller the MSE, the closer the reconstructed image is to the reference in pixel intensity and the more thoroughly the defocus energy is suppressed.

2. Normalized Mean Squared Error (NMSE);

NMSE normalizes the MSE to the energy level of the reference image, eliminating the influence of the amplitude-scale differences in the images on the evaluation results, and facilitating fair comparisons across different datasets and target categories. Its definition is:

(39) $$\mathrm{NMSE}=\frac{\sum_{i=0}^{M-1}\sum_{j=0}^{N-1}{\left[I\left(i,j\right)-K\left(i,j\right)\right]}^{2}}{\sum_{i=0}^{M-1}\sum_{j=0}^{N-1}{\left[K\left(i,j\right)\right]}^{2}}$$

The range of NMSE values is [0, +∞). The closer the value is to 0, the smaller the distortion of the reconstructed image in terms of relative energy.

3. Peak Signal-to-Noise Ratio (PSNR) [21];

PSNR is expressed in decibels (dB) to represent the signal-to-noise ratio level of the reconstructed image relative to the maximum possible signal power. The higher the PSNR, the smaller the pixel-level reconstruction error relative to the dynamic range of the image. Its definition is:

(40) $$\mathrm{PSNR}=10\cdot \log_{10}\left(\frac{L^{2}}{\mathrm{MSE}}\right)$$

Here, L represents the maximum possible value of an image pixel (typically 1 for normalized images).

4. Structural Similarity Index Measure (SSIM) [21];

SSIM comprehensively assesses the perceptual similarity between the reconstructed image and the reference image from three dimensions: luminance, contrast, and structure. Its value range is [0, 1]. For a local image window x and y, SSIM is defined as:

(41) $$\mathrm{SSIM}\left(x,y\right)=\frac{\left(2\mu_{x}\mu_{y}+C_{1}\right)\left(2\sigma_{xy}+C_{2}\right)}{\left(\mu_{x}^{2}+\mu_{y}^{2}+C_{1}\right)\left(\sigma_{x}^{2}+\sigma_{y}^{2}+C_{2}\right)}$$

Among them, $\mu_{x}$, $\mu_{y}$ are the local mean, $\sigma_{x}^{2}$, $\sigma_{y}^{2}$ are the local variance, $\sigma_{xy}$ is the local covariance, and $C_{1}={\left(K_{1}L\right)}^{2}$ and $C_{2}={\left(K_{2}L\right)}^{2}$ are the stability constants, usually taking $K_{1}=0.01$, $K_{2}=0.03$. The closer SSIM is to 1, the higher the structural consistency between the reconstructed image and the true value is, and the stronger the ability to maintain the structure of the scattering points is.

5. Image Entropy [22];

Image entropy is used to measure the uncertainty or information content of the amplitude distribution of an ISAR image. In ISAR autofocusing, the lower the entropy value, the more concentrated the image energy distribution is, and the better the focusing effect of the scattering points; conversely, the energy of the defocused image spreads along the azimuth direction, and the entropy value increases. For complex-valued ISAR images $g\left(m,n\right)$, their image entropy is defined as:

(42) $$E=-\sum_{m=1}^{M}\sum_{n=1}^{N}p_{mn}\ln p_{mn},p_{mn}=\frac{{\left|g\left(m,n\right)\right|}^{2}}{\sum_{m=1}^{M}\sum_{n=1}^{N}{\left|g\left(m,n\right)\right|}^{2}}$$

where $p_{mn}$ denotes the ratio of the energy of pixel $\left(m,n\right)$ to the total energy of the image. The lower the entropy, the more concentrated the scattering energy and the better the focusing.

6. Image Contrast [23];

Image contrast reflects the intensity difference between strong scattering points and background clutter in ISAR images and is a key indicator for measuring the target’s distinguishability. The contrast defined based on the second-order statistics of amplitude is:

(43) $$C=\frac{\sqrt{E\left\{{\left[{\left|g\left(m,n\right)\right|}^{2}-E\left\{{\left|g\left(m,n\right)\right|}^{2}\right\}\right]}^{2}\right\}}}{E\left\{{\left|g\left(m,n\right)\right|}^{2}\right\}}$$

$E\left\{\cdot \right\}$ represents the calculation of the expectation (mean value). The higher the contrast, the more prominent the energy of the strong scattering points, and the clearer the target outline and structural details. It should be noted, however, that an excessively high contrast may result from over-concentration of energy rather than from faithful structural restoration; a comprehensive assessment should therefore combine the contrast with the LGD and the entropy (see below). Furthermore, since image contrast is a no-reference indicator sensitive to local grayscale distribution and ROI selection, it is used only as an auxiliary descriptive metric rather than a sole criterion for convergence or model selection.

7. Local Gradient Dispersion (LGD) [24];

Local gradient dispersion (LGD) quantifies the spatial concentration of scattering-point energy. The LGD adopted in this paper is defined from the local gradient variance: for a well-focused ISAR image, the edges of scattering points are sharp, and the local gradient values are concentrated within a narrow range (low variance), whereas defocused images exhibit spread energy and dispersed gradient distributions (high variance). A smaller LGD therefore indicates more concentrated scattering energy and a clearer image. Letting A denote the image amplitude, the LGD is defined as

(44) $$D=\frac{1}{MN}{\sum_{m=1}^{M}\sum_{n=1}^{N}\left[\nabla A\left(m,n\right)-\bar{\nabla A}\right]}^{2}$$

Here, $\nabla A\left(m,n\right)$ represents the local gradient amplitude at the pixel $\left(m,n\right)$, while $\bar{\nabla A}$ represents the average gradient of the entire image. It should be noted that LGD is also a no-reference indicator and is therefore used as auxiliary evidence alongside visual inspection in the real-measured data evaluation.

8. Number of Parameters [25];

The number of parameters is the total count of trainable weights and biases in the network and reflects the model capacity and structural complexity; it is usually measured in millions (M). Fewer parameters imply lower storage cost and faster loading, making the network more suitable for deployment on embedded or edge devices.

9. Floating Point Operations (FLOPs) [26];

FLOPs denote the total number of floating-point operations required for a single forward pass of the network and are usually measured in gigaFLOPs (GFLOPs). As a standard metric for measuring the theoretical computational complexity of a model, FLOPs can be calculated based on the input and output feature map dimensions and the convolution kernel parameters. The smaller the GFLOPs, the lighter the theoretical computational burden of the model, and it typically corresponds to lower power consumption and higher inference throughput.

10. Inference Time per Sample (Tinf) [27];

The single-sample inference time refers to the actual time consumed by the model for a single forward pass (from input to output), measured in seconds (s). This metric is obtained under a fixed hardware platform and with a fixed input size, and directly reflects the actual operational efficiency and real-time performance of the model.

11. GPU Memory Usage (MGPU);

Memory usage refers to the peak GPU memory consumption of the model during the inference process, measured in GB. This metric encompasses the storage of model parameters, the caching of intermediate feature maps during forward propagation, and the overhead of the inference framework, reflecting the actual memory resource requirements of the model.

It should be noted that the evaluation metrics are used differently for simulated and real-measured data. For simulated data, since the ground-truth focused images are available, full-reference metrics such as MSE, NMSE, PSNR, and SSIM are used as the primary quantitative criteria. For real-measured data, ground-truth focused images are unavailable. Therefore, no-reference focus-related indicators, including image entropy, contrast, gradient-based sharpness, and local ROI profiles, are used only as auxiliary evidence. These no-reference metrics are not used as the sole criterion for model selection or convergence judgment, but rather serve to provide complementary observations together with visual inspection and scattering-profile analysis.

### 3.3. Simulation Data Performance Evaluation

To verify the refocusing performance of the proposed method, this section presents quantitative comparisons on the simulated ISAR test set of eight categories of space targets. The AFC-Mamba U-Net is compared with the complex-domain refocusing network CVPHD [13], the CNN-based baseline U-Net, and the traditional time-frequency method STFT.

**Figure 7:**
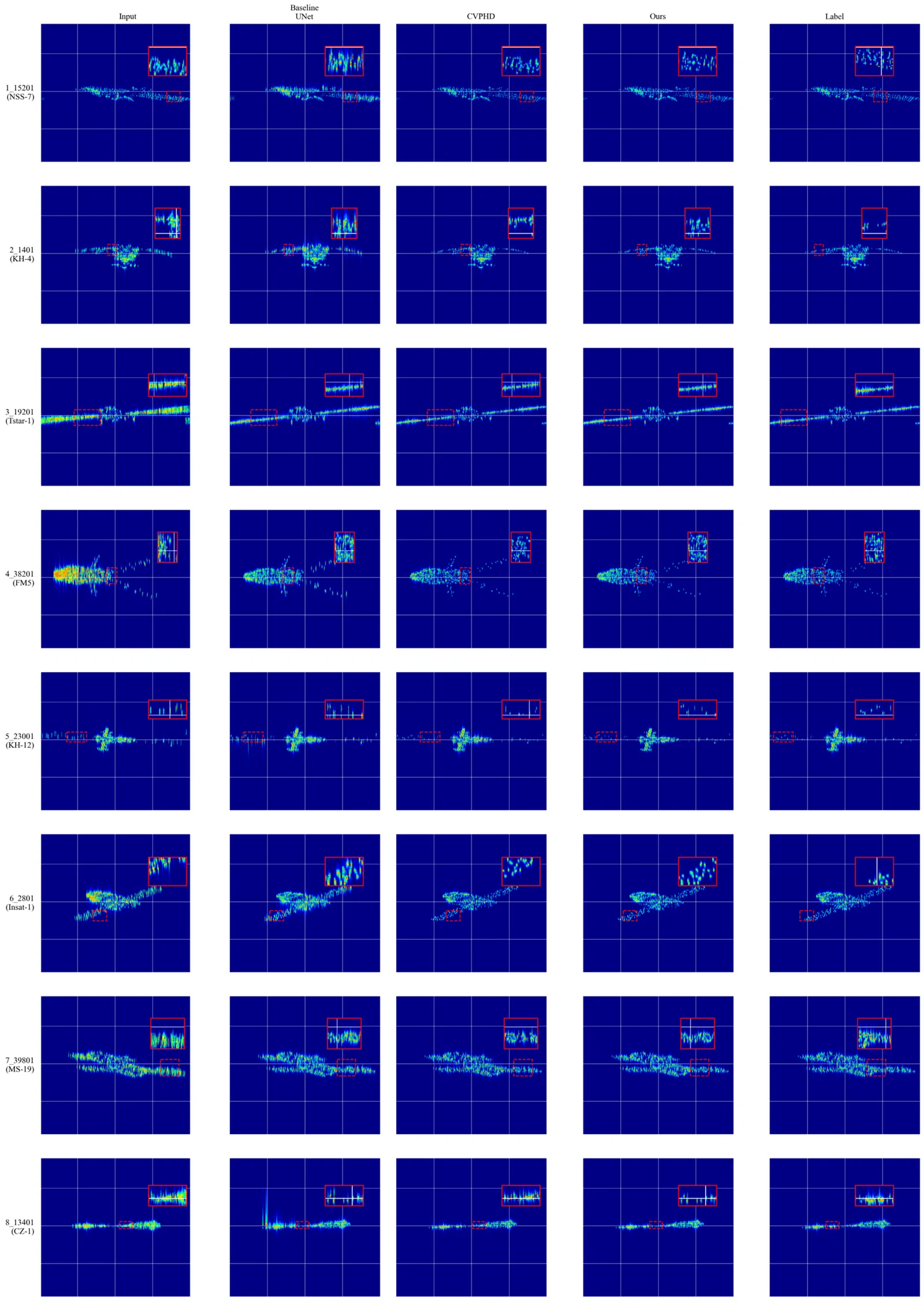
Qualitative comparison of the refocusing results of simulated ISAR images. From left to right: defocused input, baseline U-Net, CVPHD, the proposed AFC-Mamba U-Net, and the true focused amplitude image. The color intensity represents the echo amplitude, where blue indicates low amplitude and yellow/white indicates high amplitude. The red boxes indicate the zoomed-in regions for visual comparison of refocusing quality across different methods.

Figure 7 presents the qualitative comparison results of refocusing for eight typical space targets (NSS-7, KH-4, Tstar-1, FM5, KH-12, Insat-1, MS-19, CZ-1) on the simulation test set. Each row corresponds to one target category, and the columns show, from left to right, the defocused input (Input), the baseline U-Net, CVPHD, the proposed AFC-Mamba U-Net, and the ground-truth focused image (Label).

As can be seen from the magnified insets, the scattering points in the defocused inputs spread severely along azimuth, and the energy of strong and weak scattering centers is mixed together. The baseline U-Net partially suppresses the defocusing, but pronounced azimuth defocus tails remain on targets such as NSS-7, Insat-1, and MS-19. Compared with the baseline U-Net, the AFC-Mamba U-Net markedly reduces the defocusing and yields scattering structures closer to the ground truth. Nevertheless, on targets with sparser scattering distributions or more complex micro-motion components, such as Tstar-1, FM5, and MS-19, the AFC-Mamba U-Net exhibits some loss of weak scattering points or over-smoothing, resulting in a certain gap in MSE and SSIM relative to CVPHD.

Table 2 further provides a quantitative comparison of the eight target categories in terms of pixel fidelity (MSE/NMSE), structural consistency (SSIM), and energy distribution (entropy), which corroborates the qualitative observations and quantifies the relative performance gaps among the methods. The best results are highlighted in red, and the second-best results are highlighted in blue.

From the experimental results in Table 2, it can be seen that the average MSE and NMSE of the traditional time-frequency method STFT are $26.87\times 10^{-3}$ and $61.17\times 10^{-2}$. This indicates that the fixed window length limits the time-frequency resolution, making it difficult to resolve the time-varying Doppler characteristics of different scattering points of a complex maneuvering target and fundamentally restricting fine phase recovery.

The CNN-based baseline U-Net achieves an average MSE of $8.85\times 10^{-3}$ and an average NMSE of $25.52\times 10^{-3}$, with an SSIM of 0.9830. Compared with STFT, the baseline U-Net has achieved significant improvements in pixel error and structural consistency, verifying that deep learning methods have stronger feature learning and phase compensation capabilities in the ISAR refocusing task compared to traditional time-frequency methods. However, its fixed receptive field limits the modeling of long-range defocus patterns in the azimuth direction, and the gap with CVPHD is still relatively obvious.

After introducing multi-level Mamba modeling, the state bank, and the state-guided skip connection, the AFC-Mamba U-Net achieves an average MSE of $5.68\times 10^{-3}$ and an average NMSE of $14.92\times 10^{-2}$, with the SSIM increased to 0.9901. Relative to the baseline U-Net, the MSE and NMSE are reduced by approximately 35.8% and 41.5%, respectively, and the SSIM is improved by about 0.0071. This indicates that the proposed network can effectively improve the modeling ability of U-Net for ISAR long-range defocus patterns in the azimuth direction. From the perspective of category differences, AFC-Mamba U-Net maintains a high structural consistency on structures with relatively regular shapes such as NSS-7 (SSIM 0.9957) and CZ-1 (SSIM 0.9953); on targets with more sparse scattering point distributions or more complex micro-motion components such as MS-19 (NMSE 21.01) and Tstar-1 (NMSE 18.55), the gap with CVPHD is relatively large. This reflects the potential limitations of a purely real-valued Mamba architecture in fine phase compensation and sparse-scatterer correlation modeling.

Although the number of test set samples per category is small, the split is fixed (seed = 42) to ensure reproducibility. Across all eight categories, AFC-Mamba U-Net consistently outperforms baseline U-Net in MSE, NMSE, and SSIM. The cross-category standard deviation of SSIM (σ=0.0050) is 45% smaller than that of baseline U-Net (σ=0.0091), confirming that the performance gains are robust and not attributable to random measurement noise.

CVPHD achieves lower MSE and NMSE across all eight target categories, with an average MSE of 1.14 × 10^−3^ and an average NMSE of 2.37 × 10^−2^. Built on complex-valued convolution and adaptive phase compensation, CVPHD holds a clear advantage in fine echo modeling and more accurately learns the compensation mapping for the high-order phase errors introduced by maneuvering. Notably, for MS-19, CVPHD delivers the highest SSIM (0.9986) and the lowest MSE, yet its image entropy (7.93) is slightly higher than those of the baseline U-Net (7.85) and the AFC-Mamba U-Net (7.84). This phenomenon stems from the essential difference in physical meaning between the image entropy and reference-based fidelity metrics: CVPHD faithfully recovers weak scattering points and fine structural details that would otherwise be suppressed or smoothed out, which in turn raises the entropy. Therefore, for simulated data with ground-truth references, the MSE, NMSE, and SSIM serve as the core metrics that objectively quantify reconstruction fidelity, whereas the image entropy is only an auxiliary no-reference indicator of energy concentration and should not be used alone to judge focusing quality.

While CVPHD maintains outstanding refocusing accuracy on the simulated dataset, practical ISAR imaging systems impose strict constraints on real-time processing capability, memory footprint, and deployment cost. Evaluating a model solely by MSE, NMSE, and SSIM is therefore insufficient to gauge its engineering applicability. In what follows, the practical value of the AFC-Mamba U-Net is further analyzed from three perspectives: module effectiveness, real-data validation, and computational complexity.

As shown in Table 3, under the input size of 4 × 2 × 128 × 128, the parameter quantity of the AFC-Mamba U-Net is only 2.52 M, which is approximately 99.31% less than that of CVPHD (364.84 M); the GFLOPs are 6.65, which is approximately 97.55% less than that of CVPHD (271.82). In terms of inference speed, the average per-sample inference time of the AFC-Mamba U-Net is only 0.012 s (measured with a batch size of 4 and normalized by the batch size), about 1/5.4 that of CVPHD (0.065 s), corresponding to a speedup of more than 5×. In terms of memory usage, the AFC-Mamba U-Net requires only 0.11 GB, approximately 96.14% less than CVPHD (2.85 GB).

**Figure 8:**
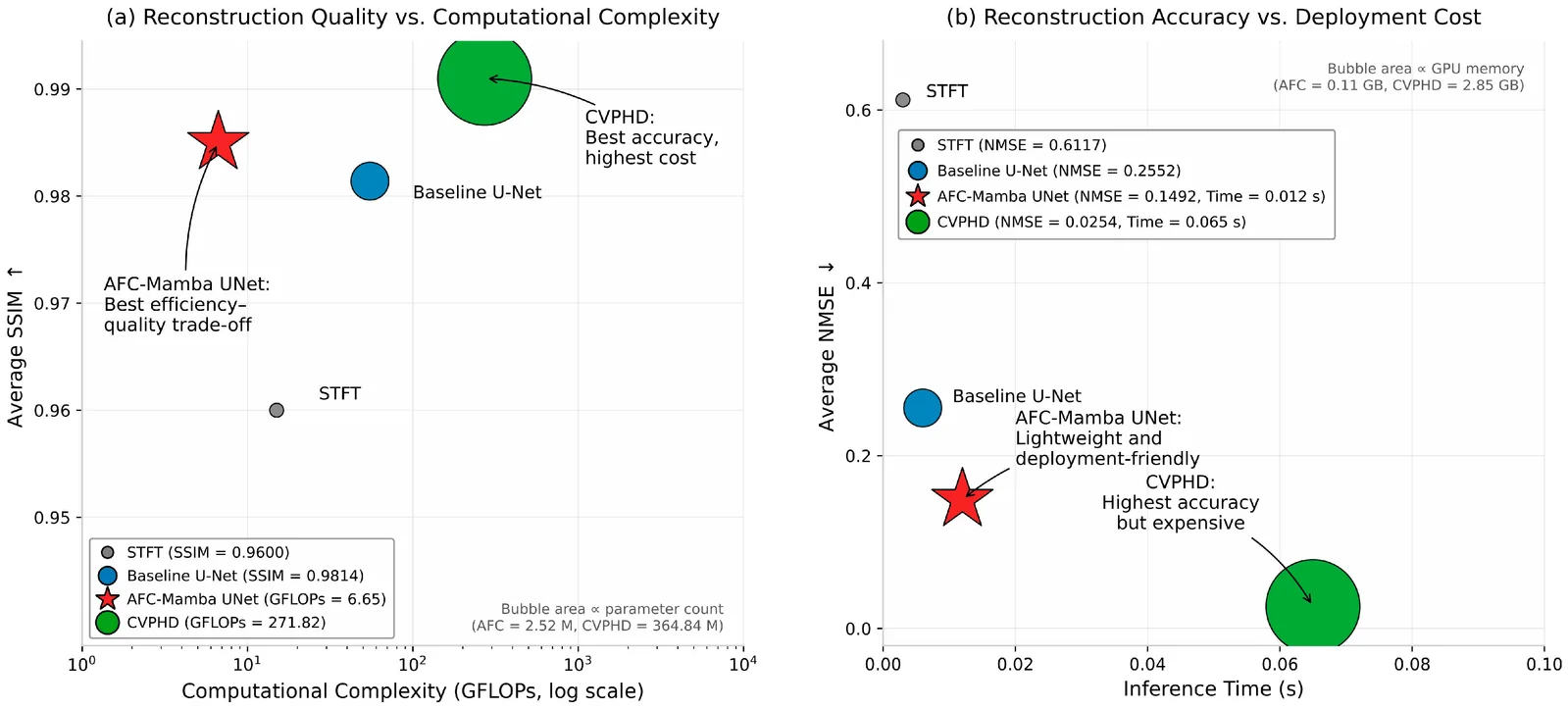
Performance and efficiency analysis of different ISAR refocusing methods. (**a**) Comparison of reconstruction quality and computational complexity. (**b**) Comparison of reconstruction accuracy and deployment cost. Bubble size in (**a**) represents the number of parameters; bubble size in (**b**) represents GPU memory usage.

To illustrate the trade-off between reconstruction quality and computational efficiency more intuitively, Figure 8 presents a performance–efficiency scatter analysis. In Figure 8a, the x-axis is the GFLOPs (logarithmic scale), the y-axis is the average SSIM, and the bubble size represents the number of parameters. CVPHD lies in the upper-right corner: although it attains the highest reconstruction accuracy, its large parameter count and high complexity make practical deployment difficult. STFT and the baseline U-Net occupy the lower-middle region, with low complexity but insufficient accuracy. The AFC-Mamba U-Net (red star) lies in the upper-left region, maintaining an average SSIM above 0.990 with only 6.65 GFLOPs and 2.52 M parameters, thus achieving the best accuracy–efficiency balance.

In Figure 8b, the x-axis is the per-sample inference time, the y-axis is the average NMSE (lower is better), and the bubble size represents the GPU memory usage. CVPHD lies in the lower-right, high-accuracy–high-cost region, whereas the AFC-Mamba U-Net lies in the lower-left region, indicating a lightweight and deployment-friendly profile. Its 0.012 s inference time and 0.11 GB memory usage verify the potential of the proposed method for real-time and embedded platforms.

Based on the above analysis, CVPHD maintains superior refocusing accuracy on both simulated and real data; however, its 364.84 M parameters and 271.82 GFLOPs can hardly meet the millisecond-level processing requirement of a real-time ISAR imaging chain. In contrast, the AFC-Mamba U-Net achieves a significant efficiency gain at an acceptable accuracy cost (the SSIM remains above 0.99 without severe degradation of structural consistency), occupying a more favorable position in the performance–efficiency trade-off. The core value of the proposed method therefore lies in the following: through multi-level state-space modeling and cross-layer feature fusion, it significantly reduces computational and storage costs while maintaining high refocusing quality, providing a lightweight and deployable solution for the practical application and real-time processing of ISAR refocusing.

### 3.4. Ablation Experiments and Convergence Analysis

#### 3.4.1. Module Ablation Experiment

To verify the effectiveness of each proposed component, we conduct ablation experiments by systematically removing or replacing components from the full model. The results are summarized in Table 4. Note that the ablation experiments are performed on a validation subset (50 patches) with a patch-level independent min–max normalization protocol, whereas the quantitative comparison in Table 2 is performed on an independent test set (full-size images) with a whole-image unified evaluation protocol. The absolute values in Table 2 and Table 4 are therefore not directly comparable; Table 4 is used only to assess the relative contribution of each module under identical experimental settings, and its trends are consistent with the whole-image test. The best results are shown in bold.

The baseline adopts a purely convolutional U-Net and achieves a PSNR of 41.99 dB, an SSIM of 0.9885, and an NMSE of 0.0747. The results show that local convolution recovers part of the scattering-point details but, constrained by its fixed receptive field, struggles to model the long-range azimuth defocus patterns caused by maneuvering.

Introducing the Bottleneck Mamba into the baseline model (Model_B) improves the PSNR by 0.85 dB and reduces the NMSE by approximately 8.8%. This indicates that embedding the Mamba state-space model at the deepest layer of the network can effectively expand the global receptive field, enhance global context modeling capability, and is particularly helpful for capturing long-range defocusing features distributed along the azimuth direction.

Further introducing Mamba at the third encoder stage (Model C) raises the PSNR to 43.11 dB and reduces the NMSE to 0.0619. Compared with Model B, this configuration adds azimuth long-range dependency modeling at the deeper encoding stage, complements the global representation of the bottleneck, and further improves the recovery accuracy of scattering-point focusing structures.

However, when continuing to introduce Mamba at the second encoder stage (Enc2) (Model_D), the NMSE further decreases to 0.0592, but the PSNR and SSIM drop to 42.92 dB and 0.9892, respectively. This phenomenon indicates that although introducing global modeling at the middle-high-resolution encoding layer helps improve the overall energy distribution, excessively expanding the receptive field may smooth high-frequency local details or cause the network to over-focus on global defocusing patterns while weakening the preservation capability of fine structures.

After introducing the state bank module (Model_E), the PSNR significantly improves to 43.29 dB, indicating that multi-scale state feature fusion can effectively enhance the network’s representation capability for defocusing states. However, its SSIM drops to 0.9860, indicating that relying solely on state aggregation for multi-scale information fusion may introduce structural perturbations, causing the network to sacrifice some local structure preservation capability while optimizing overall energy recovery.

To verify that the gain of the state-guided skip connection specifically arises from phase-state guidance rather than from simply introducing a gating layer, we replace the SGSC with a standard attention gate (Model F) and a simple encoder-only gated skip connection (Model G). As shown in Table 4, Model G attains an SSIM of only 0.9871, indicating that local encoder-only gating lacks the global structural context required for fine-detail preservation. The standard attention gate (Model F) improves the PSNR to 43.04 dB, yet its SSIM remains at 0.9888, indicating that spatial attention alone cannot compensate for the structural perturbations introduced by multi-scale state fusion. Only the full model with the SGSC improves all three metrics simultaneously, demonstrating that the bottleneck state provides indispensable global guidance beyond generic gating.

The final full model (Model_H_Full) achieves optimal values on all three metrics: PSNR, SSIM, and NMSE, at 43.30 dB, 0.9903, and 0.0541, respectively. Compared with the baseline model, the PSNR improves by 1.31 dB and the NMSE decreases by 27.6%; compared with Model_E, the PSNR remains stable (43.29 dB to 43.30 dB), but the SSIM significantly recovers from 0.9860 to 0.9903, and the NMSE further decreases from 0.0598 to 0.0541. Compared with Models F and G, the SGSC achieves superior performance across all metrics, which verifies the effectiveness of the state-guided skip connection: by dynamically screening the encoder skip features with the bottleneck state, it effectively suppresses the propagation of defocus artifacts to the decoder while preserving key scattering-point details and spatial structures.

The above ablation study systematically verifies the effectiveness and complementarity of each module: the bottleneck and encoder Mambas jointly enhance the modeling of long-range azimuth dependencies; the state bank provides a multi-scale defocus-state representation; and the state-guided skip connections improve structural consistency and reduce the overall reconstruction error through state-guided feature selection. The full network achieves the best performance on all metrics, fully demonstrating the rationality of the proposed architecture and the synergistic gains among the modules.

#### 3.4.2. Loss-Term Ablation Experiment

To verify the contribution of each loss component in the jointly supervised objective, we conduct loss-term ablation experiments and sensitivity analysis on the same validation subset (50 patches) under identical training settings. The results are summarized in Table 5 and Table 6.

Table 5 reports the loss-term ablation results on the validation subset. The full joint loss achieves the best overall performance, with a PSNR of 43.30 dB, an NMSE of 0.0541, and an SSIM of 0.9903, indicating that the adopted loss components are complementary. Removing the gradient loss decreases the PSNR from 43.30 dB to 42.63 dB and increases the NMSE from 0.0541 to 0.0640, demonstrating its importance in preserving local structures and scattering-center boundaries. Without the logarithmic amplitude loss, the PSNR drops to 42.76 dB, and the NMSE increases to 0.0572, suggesting that this term helps balance the large dynamic range between strong and weak scatterers. Removing the dual-domain loss causes a mild performance degradation, with the PSNR decreasing to 43.14 dB and the NMSE increasing to 0.0583, which indicates that it mainly serves as an auxiliary constraint for global spectral consistency. The most evident degradation is observed when the scattering loss is removed. In this case, the NMSE increases from 0.0541 to 0.0915, and the SSIM decreases from 0.9903 to 0.9851, showing that this term is effective in suppressing energy spreading and preserving focused scattering centers. Overall, the ablation results verify that the proposed joint loss provides a balanced supervision mechanism, where the main spatial-domain losses ensure reconstruction fidelity, while the auxiliary losses improve dynamic-range stability, spectral consistency, and focusing clarity.

To further justify the specific weight values, we conduct a sensitivity study by perturbing each coefficient around its nominal value while fixing the others. As shown in Table 6, the primary terms ($\lambda_{mag}$, $\lambda_{grad}$) tolerate ±20% variation with negligible impact (PSNR < 0.15 dB fluctuation), and the auxiliary terms remain insensitive within [0.05, 0.2]. This confirms that the hierarchical weighting is not sensitive to minor tuning and reflects a robust design choice.

#### 3.4.3. Convergence Analysis

**Figure 9:**
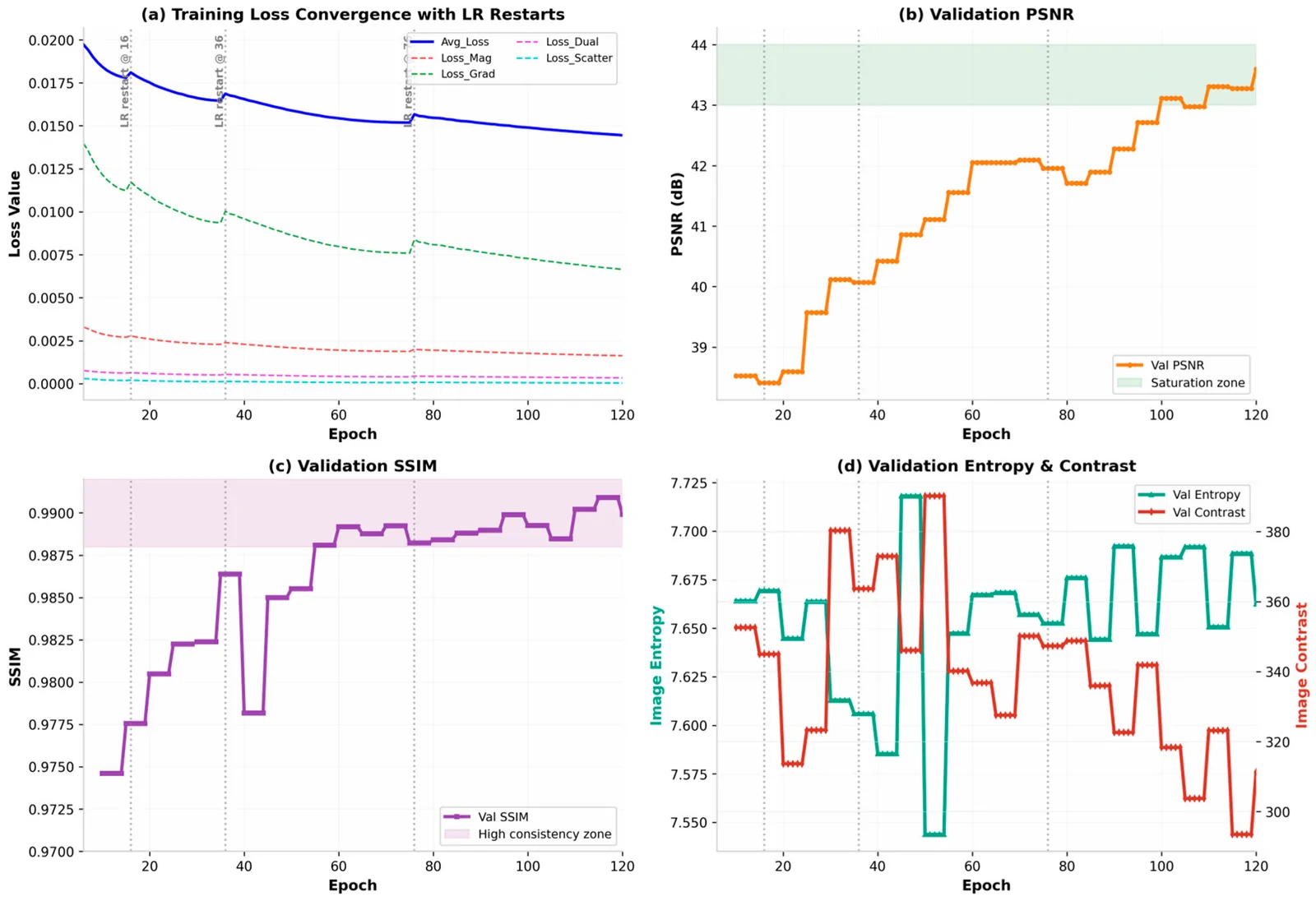
Training dynamics of the AFC-Mamba U-Net over 120 epochs. (**a**) Convergence curve of the training loss; (**b**–**d**) validation metrics: PSNR, SSIM, image entropy, and contrast. The vertical dashed lines in (**a**) indicate the learning rate restart points at epochs 16, 36, and 76. The green-shaded area in (**b**) marks the PSNR saturation region (>43 dB), and the purple-shaded area in (**c**) marks the high structural-consistency region (SSIM > 0.988).

To further verify the rationality of the training strategy, Figure 9 presents the complete training dynamics of AFC-Mamba U-Net over 120 epochs. Specifically, Figure 9a shows the convergence curve of the training loss, while Figure 9b–d depict the evolution curves of PSNR, SSIM, image entropy, and contrast on the validation set.

As shown in Figure 9a, the total loss (Avg_Loss) steadily decreases from an initial 0.0197 to 0.0145, with the gradient loss (Loss_Grad) contributing the most and continuing to decrease, indicating that the network’s edge-recovery capability for defocused images continuously strengthens with training. The amplitude loss (Loss_Mag) and logarithmic loss (Loss_Log) converge synchronously, ensuring the stability of reconstructed amplitude and dynamic range. The dual-domain loss (Loss_Dual) and scattering loss (Loss_Scatter) remain at low levels, indicating that no gradient conflict occurs between the frequency-domain and spatial-domain branches. The vertical dashed lines in the figure mark the positions of three learning rate restarts (LR Restart) (at epochs 16, 36, and 76). After each restart, the total loss briefly rises and then continues to decrease, verifying that the cosine-annealing restart strategy helps the training escape local optima.

Regarding validation set metrics, Figure 9b shows that the PSNR gradually climbs from 38.5 dB to 43.6 dB and enters the saturation region (green-shaded area) after epoch 100, indicating that the network’s pixel-level reconstruction accuracy is approaching convergence. Figure 9c shows that the SSIM rapidly rises from 0.975 to above 0.990 and stabilizes in the 0.988–0.991 range (purple-shaded area) in the later stages, indicating that multi-level Mamba modeling and the state-guided skip mechanism effectively enhance cross-layer structure preservation capability. It should be noted that in Figure 9d, image entropy (Entropy) and contrast (Contrast) exhibit opposite fluctuation trends in the later training stages: entropy stabilizes between 7.60 and 7.70, while contrast oscillates within the 300–380 range. Although entropy and contrast are commonly used as no-reference focus-related indicators in radar image evaluation, they are sensitive to local grayscale fluctuations, ROI selection, and minor parameter updates. Therefore, entropy and contrast are not used for early stopping, checkpoint selection, or convergence judgment in this work. The convergence behavior and the final model selection are determined by the supervised validation loss and the full-reference metrics, including MSE, NMSE, PSNR, and SSIM, on the simulated validation set. Entropy and contrast are reported only to describe the evolution of image gray-level distribution during training. For real-measured data, entropy and contrast are reported only as auxiliary descriptive indicators and are interpreted jointly with visual comparisons, ROI intensity distributions, and scattering-center preservation analysis.

### 3.5. Verification with Actual Measurement Data

To examine the generalization capability of the proposed method in real-world scenarios, we conducted experiments on a representative frame from real ISAR data of the Yak-42 aircraft. It should be emphasized that the ground-truth focused image is unavailable for real-measured data; therefore, the following quantitative results are interpreted as no-reference auxiliary evidence rather than absolute full-reference performance verification. In this section, image entropy, contrast, and LGD, among other no-reference indicators, are used only as complementary descriptive metrics alongside visual inspection and spatial profile analysis. The results are shown in Table 7.

From the perspective of background clutter suppression, the traditional STFT method reduces the image entropy from 8.4620 to 7.2358 and improves the contrast from 161.13 to 194.22, indicating that the fixed-window time-frequency analysis suppresses background energy to a certain extent. However, its LGD increases from 0.000469 to 0.000575, indicating that the window-length-limited time-frequency resolution cannot resolve the time-varying Doppler components of a maneuvering target; consequently, the scattering-point energy spreads along the azimuth, and the focusing clarity of the target scattering structure degrades. This demonstrates that traditional fixed-parameter time-frequency methods have inherent limitations for real ISAR data of complex maneuvering targets.

The baseline U-Net yields the lowest entropy (6.8883) and the highest contrast (305.09), yet its LGD (0.000400) is significantly higher than that of AFC-Mamba (0.000084). Combined with its lower SSIM on simulated data, this indicates that the baseline U-Net merely reallocates defocused energy into a few strong peaks via local convolution, producing a “false focusing” with high contrast but poor structural fidelity. This reveals a clear cross-domain generalization bottleneck of pure CNNs.

**Figure 10:**
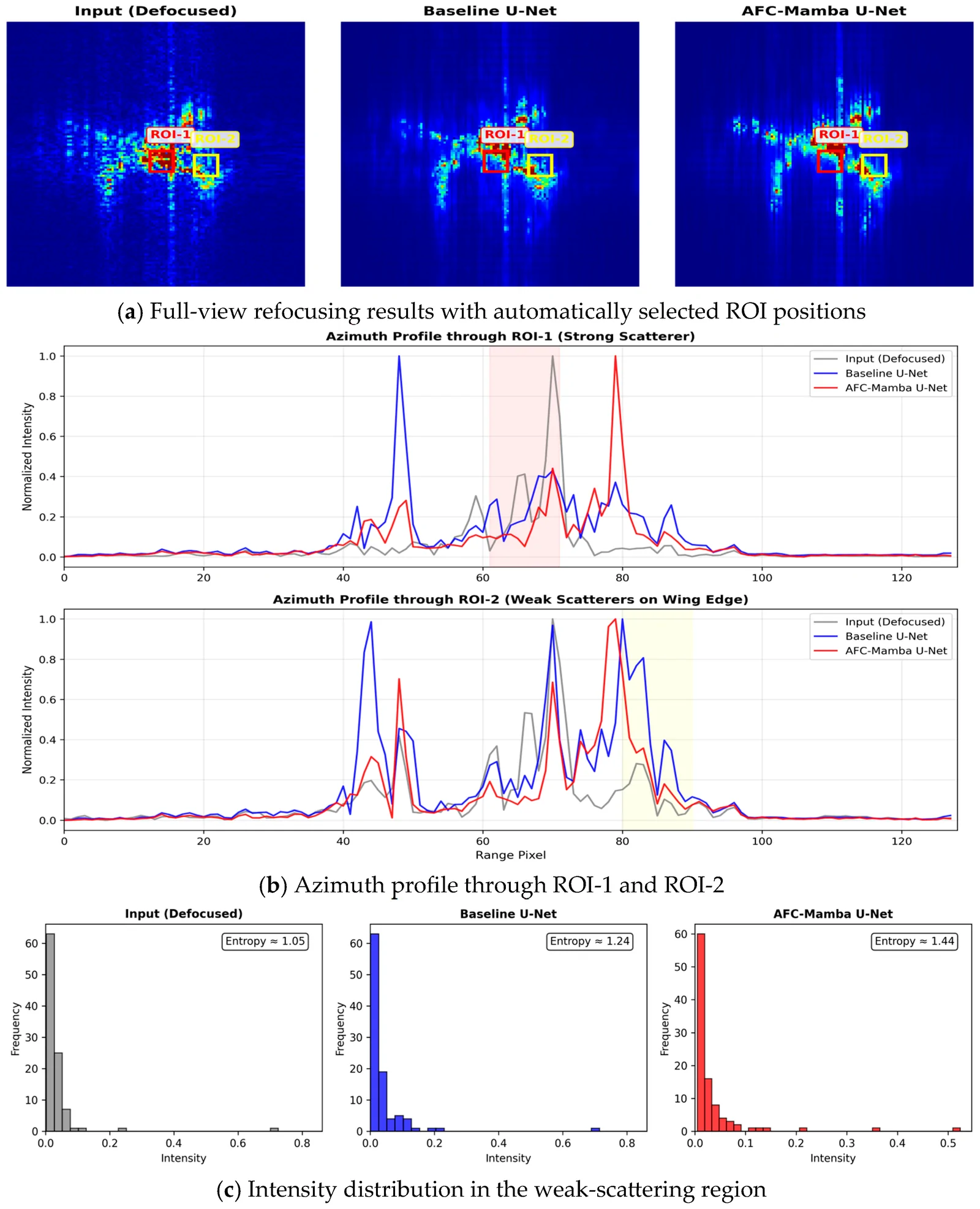
Localized analysis of strong and weak-scattering regions on the Yak-42 data. (**a**) Full-view results with ROI positioning (ROI-1: strong scatterer in red; ROI-2: wing-edge weak scatterer in yellow). (**b**) Normalized azimuth profiles through ROI-1 (top) and ROI-2 (bottom). Gray, blue, and red curves represent the input (defocused), Baseline U-Net, and AFC-Mamba U-Net, respectively. In ROI-2, AFC-Mamba preserves multi-peak fluctuations consistent with the input, while baseline over-smooths weak structures. (**c**) Intensity histograms within ROI-2, showing AFC-Mamba has the broadest distribution (entropy ≈ 1.44) versus baseline (entropy ≈ 1.24) and input (entropy ≈ 1.05).

In contrast, the proposed AFC-Mamba U-Net reduces the image entropy from 8.4620 to 8.1624 and increases the contrast from 161.13 to 206.08, corresponding to a relative contrast improvement of approximately 27.9%. In addition, the proposed method obtains the lowest LGD value among the compared methods. These observations suggest that the proposed method tends to enhance the focusing clarity of dominant scattering centers while maintaining weak-scattering structures. However, considering the volatility of no-reference metrics, the real-data comparison is not based solely on entropy or contrast. The azimuth profiles through ROI-1 and ROI-2 and the intensity distribution in weak-scattering regions further show that the proposed method suppresses azimuthal defocusing smears without excessively eliminating weak scatterers. Therefore, the real-measured experiment is used as a complementary generalization demonstration, while the primary quantitative validation is conducted on the simulated dataset with available ground truth. To further examine the preservation of weak scatterers, pixel-level analysis using automatically selected ROIs is presented in Figure 10.

In Figure 10a, ROI-1 covers a strong-scatterer region and ROI-2 a weak-scatterer region. The 1-D azimuth profiles show that, in the weak-scattering region (ROI-2), the AFC-Mamba U-Net retains the multi-peak fluctuations of the defocused input, whereas the baseline U-Net tends to suppress weak-scatterer structures or generate spurious isolated peaks. The intensity histogram of ROI-2 further explains the entropy values in Table 7: AFC-Mamba exhibits the widest distribution (local entropy ≈ 1.44), indicating that multiple weak-scattering intensity levels are preserved, while the baseline (local entropy ≈ 1.24) and the defocused input (local entropy ≈ 1.05) show more concentrated distributions. This confirms that the higher global entropy of AFC-Mamba reflects richer structural details rather than noise.

In comparison, CVPHD achieves lower entropy (6.4980) and higher contrast (363.03). The joint behavior of low entropy, high contrast, and competitive LGD suggests that CVPHD achieves deep focusing on this representative frame, consistent with its performance on simulated data. The real-data experiments show that although the AFC-Mamba U-Net still has a gap with CVPHD in absolute focusing depth, it obtains more favorable structure preservation and focusing characteristics compared with STFT and the baseline U-Net, contributing to improved target discriminability of defocused images. Combined with the complexity analysis in Section 3.3, it can be seen that the proposed method significantly reduces computational and storage overhead while maintaining high refocusing quality, making it more suitable for deployment on real-time or embedded platforms in real-world scenarios; the high complexity of CVPHD limits its engineering practicality, while STFT and the CNN-based U-Net have inherent limitations in focusing accuracy or structure preservation.

**Figure 11:**
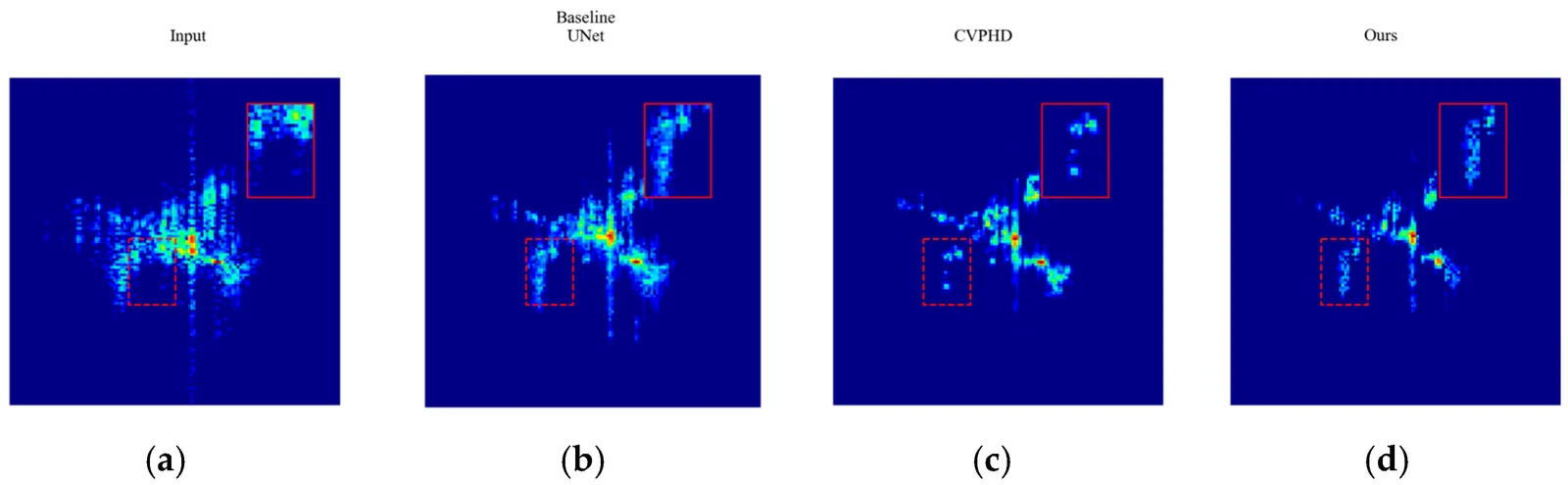
The refocused results of the real ISAR images of the Yak-42 aircraft. (**a**) Defocused input, (**b**) baseline U-Net, (**c**) CVPHD, (**d**) the proposed AFC-Mamba U-Net. The color intensity represents the echo amplitude, where blue indicates low amplitude and yellow/white indicates high amplitude. The red boxes mark the regions of interest (ROIs) for visual comparison.

Figure 11 presents a qualitative comparison of refocusing on real ISAR data of the Yak-42 aircraft. In the input image, scattering points are severely defocused along the azimuth direction. The baseline U-Net achieves partial focusing, but residual defocus is still visible. CVPHD achieves good overall focusing with clear scattering centers, but some local weak scattering points are missing. The AFC-Mamba U-Net maintains clear scattering centers while preserving a more complete distribution of weak scattering points, resulting in higher overall target discriminability.

## 4. Conclusions

This paper proposes an Azimuth-Frequency Conditioned Mamba U-Net (AFC-Mamba U-Net) for ISAR image refocusing. Introducing state-space models into the encoder–decoder architecture effectively alleviates the problems of limited receptive fields in traditional CNNs and excessively high computational complexity in Transformers. The designed AFCMambaBlock captures long-range azimuth defocusing dependencies with linear complexity, the phase-state bottleneck aggregates multi-scale global state representations, and the state-guided skip connection further utilizes this representation to dynamically suppress the propagation of defocusing artifacts.

Experiments on a simulated dataset containing eight classes of space targets and real Yak-42 aircraft data show that the proposed method achieves a significant improvement in refocusing quality compared with the CNN-based U-Net. The complex-valued network CVPHD still achieves the best absolute reconstruction fidelity on simulated data. However, while maintaining high structural consistency (SSIM > 0.99), the method proposed in this paper reduces the model parameter count and computational complexity by approximately two orders of magnitude, achieves over 5-fold speedup in inference, and cuts GPU memory usage by around 96%, providing a lightweight and viable technical solution for real-time and embedded deployment of ISAR refocusing.

Future work will focus on two directions: first, exploring complex state-space models to enable the network to operate directly in the complex domain, thereby further improving phase compensation accuracy; second, designing adaptive state scanning mechanisms for joint range-azimuth degradation and complex non-stationary maneuvering, expanding the applicability of the method under more extreme imaging conditions.

## Figures and Tables

**Table 1:** Imaging parameters of the simulated ISAR dataset.

Parameter	Value
Bandwidth	240 MHz
Carrier frequency	5.4 GHz
Rotation angle	−1.92~1.92°
Target rotation speed	0.1 rad/s
Target rotation acceleration	5000 rad/s^2^
Number of pixels in the image	128 × 128

**Table 2:** Quantitative comparison of simulated ISAR datasets.

Model	Method	MSE (×10^−3^)	NMSE (×10^−2^)	SSIM	Entropy
NSS-7	STFT	7.67	33.29	0.9774	7.00
	Baseline U-Net	4.87	28.20	0.9630	6.96
	AFC-Mamba U-Net	2.21	8.66	0.9957	6.94
	CVPHD	0.23	0.95	0.9995	6.92
KH-4	STFT	28.39	62.93	0.9648	7.43
	Baseline U-Net	12.16	30.41	0.9880	7.44
	AFC-Mamba U-Net	6.26	13.65	0.9922	7.41
	CVPHD	1.27	2.76	0.9991	7.40
Tstar-1	STFT	28.80	60.12	0.9540	7.83
	Baseline U-Net	10.21	22.69	0.9856	7.81
	AFC-Mamba U-Net	9.05	18.55	0.9864	7.80
	CVPHD	1.63	3.43	0.9982	7.71
FM5	STFT	33.58	72.41	0.9432	8.00
	Baseline U-Net	8.64	19.35	0.9868	7.95
	AFC-Mamba U-Net	8.59	17.14	0.9876	7.95
	CVPHD	2.21	4.63	0.9978	7.95
KH-12	STFT	29.10	63.13	0.9717	7.30
	Baseline U-Net	7.80	17.27	0.9917	7.29
	AFC-Mamba U-Net	6.82	14.40	0.9923	7.26
	CVPHD	0.91	1.95	0.9993	7.27
Insat-1	STFT	25.70	63.89	0.9522	7.65
	Baseline U-Net	13.26	35.13	0.9790	7.65
	AFC-Mamba U-Net	5.13	12.35	0.9907	7.58
	CVPHD	0.83	2.03	0.9991	7.63
MS-19	STFT	32.30	64.87	0.9369	7.99
	Baseline U-Net	6.47	33.93	0.9750	7.85
	AFC-Mamba U-Net	1.48	21.01	0.9804	7.84
	CVPHD	0.72	1.46	0.9986	7.93
CZ-1	STFT	29.42	68.72	0.9797	7.11
	Baseline U-Net	7.36	17.19	0.9820	7.11
	AFC-Mamba U-Net	5.91	13.61	0.9953	7.10
	CVPHD	1.34	3.11	0.9993	7.09

**Table 3:** Comparison of computational efficiency. The inference time is reported as the average per-sample time within a batch of 4 (input: 4 × 2 × 128 × 128).

Method	Parameter Quantity (M)	GFLOPs	Time (s)	GPU Memory (GB)
CVPHD	364.84	271.82	0.065	2.85
AFC-Mamba U-Net	2.52	6.65	0.012	0.11

**Table 4:** Ablation results on the simulated ISAR test set.

Configuration	PSNR	NMSE	SSIM
Baseline_A (CNN U-Net)	41.99	0.0747	0.9885
Model_B (+Bottleneck Mamba)	42.84	0.0681	0.9898
Model_C (+Enc3 Mamba)	43.11	0.0619	0.9898
Model_D (+Enc2 Mamba)	42.92	0.0592	0.9892
Model_E (+State Bank)	43.29	0.0598	0.9860
Model_F (Attention Gate Skip)	43.04	0.0642	0.9888
Model_G (Simple Gated Skip)	41.90	0.0680	0.9871
Model_H_Full (+State-Guided Skip)	**43.30**	**0.0541**	**0.9903**

**Table 5:** Ablation study of different loss terms on the validation subset.

Configuration	$\lambda_{grad}$	$\lambda_{log}$	$\lambda_{dual}$	$\lambda_{scatter}$	PSNR	NMSE	SSIM
Loss_Full	0.3	0.5	0.1	0.01	43.30	0.0541	0.9903
Loss_without_grad	0	0.5	0.1	0.01	42.63	0.0640	0.9890
Loss_without_lop	0.3	0	0.1	0.01	42.76	0.0572	0.9900
Loss_without_dual	0.3	0.5	0	0.01	43.14	0.0583	0.9897
Loss_without_scatter	0.3	0.5	0.1	0	42.98	0.0915	0.9851

**Table 6:** Sensitivity analysis of the loss weights on the simulated ISAR validation subset.

Configuration	$\lambda_{mag}$	$\lambda_{grad}$	$\lambda_{\log}$	$\lambda_{dual}$	$\lambda_{\mathrm{scatter}}$	PSNR	NMSE	SSIM
Nominal	1.0	0.3	0.5	0.1	0.01	43.30	0.0541	0.9903
$\lambda_{mag}$ × 0.8	0.8	0.3	0.5	0.1	0.01	42.95	0.0570	0.9896
$\lambda_{mag}$ × 1.2	1.2	0.3	0.5	0.1	0.01	42.88	0.0572	0.9898
$\lambda_{grad}$ × 0.8	1.0	0.24	0.5	0.1	0.01	43.15	0.0555	0.9900
$\lambda_{grad}$ × 1.2	1.0	0.36	0.5	0.1	0.01	43.22	0.0548	0.9901
$\lambda_{\log}$ × 0.2	1.0	0.3	0.1	0.1	0.01	43.05	0.0568	0.9879
$\lambda_{\log}$ × 2.0	1.0	0.3	1.0	0.1	0.01	42.98	0.0575	0.9806
$\lambda_{dual}$ × 0.5	1.0	0.3	0.5	0.05	0.01	43.24	0.0548	0.9901
$\lambda_{dual}$ × 2.0	1.0	0.3	0.5	0.3	0.01	43.18	0.0552	0.9853
$\lambda_{\mathrm{scatter}}$ × 0.5	1.0	0.3	0.5	0.1	0.005	43.02	0.0720	0.9878
$\lambda_{\mathrm{scatter}}$ × 2.0	1.0	0.3	0.5	0.1	0.02	43.25	0.0545	0.9902

**Table 7:** Quantitative results of real ISAR data (Yak-42 aircraft).

Index	Input (Defocused)	STFT	Baseline U-Net	AFC-Mamba U-Net	CVPHD
Entropy	8.4620	7.2358	6.8883	8.1624	6.4980
LGD	0.000469	0.000575	0.0004	0.000084	0.000353
Contrast	161.13	194.22	305.09	206.08	363.03

## Data Availability

The simulated dataset used in this study was generated based on parameters from publicly available literature. The real Yak-42 data were provided by partner institutions and are not publicly available due to data sharing agreement restrictions. The relevant code will be open-sourced upon acceptance of the paper.

## References

[1] Muñoz-Ferreras J.M., Pérez-Martínez F. (2008). Uniform Rotational Motion Compensation for Inverse Synthetic Aperture Radar with Non-Cooperative Targets. IET Radar Sonar Navig..

[2] Chen V.C., Qian S. (1998). Joint Time-Frequency Transform for Radar Range-Doppler Imaging. IEEE Trans. Aerosp. Electron. Syst..

[3] Berizzi F., Mese E.D., Diani M., Martorella M. (2001). High-Resolution ISAR Imaging of Maneuvering Targets by Means of the Range Instantaneous Doppler Technique: Modeling and Performance Analysis. IEEE Trans. Image Process..

[4] Chen X., Guan J., Liu N., He Y. (2014). Maneuvering Target Detection via Radon-Fractional Fourier Transform-Based Long-Time Coherent Integration. IEEE Trans. Signal Process..

[5] Peleg S., Porat B. (1989). Estimation and Classification of Signals with Polynomial Phase. The Sixteenth Conference of Electrical and Electronics Engineers in Israel.

[6] Wang H., Chen Y., Zhang Y., Luo Y., Zhang Q. (2023). High-Resolution ISAR Imaging Method for Maneuvering Targets Based on a Hybrid Transformer. IEEE Trans. Antennas Propagat..

[7] Sun G., Zhang F. (2020). Convolutional Neural Network (CNN)-Based Fast Back Projection Imaging with Noise-Resistant Capability. IEEE Access.

[8] Ning Q., Wang H., Yan Z., Wang Z., Lu Y. (2024). A Fast and Robust Range Alignment Method for ISAR Imaging Based on a Deep Learning Network and Regional Multi-Scale Minimum Entropy Method. Remote Sens..

[9] Hu C., Wang L., Li Z., Sun L., Loffeld O. (2019). Inverse Synthetic Aperture Radar Imaging Using Complex-value Deep Neural Network. J. Eng..

[10] Gao J., Deng B., Qin Y., Wang H., Li X. (2019). Enhanced Radar Imaging Using a Complex-Valued Convolutional Neural Network. IEEE Geosci. Remote Sens. Lett..

[11] Gennarelli G., Esposito G., Soldovieri F. (2025). Analysis of U-NET Super-Resolving Capabilities in Radar Imaging. IEEE Sens. J..

[12] Tang H., Li Z., Zhang D., He S., Tang J. (2025). Divide-and-Conquer: Confluent Triple-Flow Network for RGB-T Salient Object Detection. IEEE Trans. Pattern Anal. Mach. Intell..

[13] Yuan H., Li H., Zhang Y., Wang Y., Liu Z., Wei C., Yao C. (2022). High-Resolution Refocusing for Defocused ISAR Images by Complex-Valued Pix2pixHD Network. IEEE Geosci. Remote Sens. Lett..

[14] Xiao Y., Yuan Q., Jiang K., Chen Y., Zhang Q., Lin C.-W. (2024). Frequency-Assisted Mamba for Remote Sensing Image Super-Resolution. IEEE Trans. Multimed..

[15] Gu A., Dao T. (2023). Mamba: Linear-Time Sequence Modeling with Selective State Spaces. arXiv.

[16] Liu X., Zhang C., Huang F., Xia S., Wang G., Zhang L. (2026). Vision Mamba: A Comprehensive Survey and Taxonomy. IEEE Trans. Neural Netw. Learn. Syst..

[17] Guo H., Li J., Dai T., Ouyang Z., Ren X., Xia S.-T. (2024). MambaIR: A Simple Baseline for Image Restoration with State-Space Model. European Conference on Computer Vision.

[18] Zou Z., Yu H., Huang J., Zhao F. (2024). FreqMamba: Viewing Mamba from a Frequency Perspective for Image Deraining. Proceedings of the 32nd ACM International Conference on Multimedia.

[19] Zhu B., Zhu W., Pang H., Tong F., Qu W., Li C. (2026). Multichannel ROI Joint Azimuth Compression Algorithm for InISAR Imaging Under Space-Time Varying Doppler. IEEE Trans. Aerosp. Electron. Syst..

[20] Zhu B., Zhu W., Li C., Qu W., Qui L. (2025). ISAR Imaging with 3-D Large Angles Using Nonuniform Polar Format and FrFT Segmentation Compensation. IEEE Trans. Geosci. Remote Sens..

[21] Wang Z., Bovik A.C., Sheikh H.R., Simoncelli E.P. (2004). Image Quality Assessment: From Error Visibility to Structural Similarity. IEEE Trans. Image Process..

[22] Li X., Liu G., Ni J. (1999). Autofocusing of ISAR Images Based on Entropy Minimization. IEEE Trans. Aerosp. Electron. Syst..

[23] Martorella M., Berizzi F., Haywood B. (2005). Contrast Maximisation Based Technique for 2-D ISAR Autofocusing. IEE Proc. Radar Sonar Navig..

[24] Pertuz S., Puig D., Garcia M.A. (2013). Analysis of Focus Measure Operators for Shape-from-Focus. Pattern Recognit..

[25] Yang Q., Zhang Y., Chen Y., Zhang J., Zhang S. (2025). SSPFusion: A Semantic Structure-Preserving Approach for Infrared and Visible Image Fusion. arXiv.

[26] Hu Z.Z., Chen X., Chung V.Y.Y., Shen Y. (2025). Beyond Subspace Isolation: Many-to-Many Transformer for Light Field Image Super-Resolution. IEEE Trans. Multimed..

[27] Wang S., Wang Y., Hu B., Zhang L., Zhang X.-P., Wei M. (2026). M2IR: Proactive All-in-One Image Restoration via Mamba-Style Modulation and Mixture-of-Experts. arXiv.

